# Sexual Orientation Affects Neural Responses to Subtle Social Aggression Signals

**DOI:** 10.1007/s10508-023-02661-z

**Published:** 2023-07-27

**Authors:** Katrin T. Lübke, Dunja Storch, Bettina M. Pause

**Affiliations:** Department of Experimental Psychology, Heinrich-Heine-University Düsseldorf, Universitätsstr. 1, 40225 Düsseldorf, Germany

**Keywords:** Aggression, Body odors, Chemosensory communication, Sexual orientation, ERP

## Abstract

The current series of studies are the first to examine brain responses to social aggression signals as a function of male and female sexual orientation. For the first set of studies (1a, 1b), axillary sweat had been collected from 17 heterosexual men and 17 heterosexual women aggressively responding to frustrating opponents (aggression condition) and while playing a construction game (control condition). Sweat samples were pooled according to sex and condition, and presented via a constant flow olfactometer to 17 gay and 23 heterosexual men (Study 1a), and 19 lesbian and 25 heterosexual women (Study 1b). Ongoing EEG was recorded from 61 scalp locations, chemosensory event-related potentials (CSERPs; P2, P3-1, P3-2) were analyzed, and neuronal sources calculated (low resolution electromagnetic tomography). Within the second set of studies (2a, 2b), pictures of males’ and females’ weak angry and neutral facial expressions were presented to 21 gay and 23 heterosexual men (Study 2a), and 19 lesbian and 26 heterosexual women (Study 2b), and ERPs (N170, P3) were analyzed. Gay men showed larger P3-1 amplitudes than heterosexual men upon presentation of male aggression sweat, accompanied by activation of the right inferior frontal gyrus (IFG, BA 10). Gay men also displayed longer N170 latencies in response to men’s compared to women’s angry facial expressions, while heterosexual men did not. In women, sexual orientation did not affect the processing of aggression sweat or anger expressions. Gay men showed preferential processing of chemosensory aggression signals (P3-1 amplitudes), indicating fine-tuned socioemotional sensitivity, related to activation of brain areas involved in emotion regulation (IFG). They further process the relative relevance of visual aggression signals (N170 latency). These results were in line with theories proposing a common evolutionary pathway for same-sex attraction and traits easing social integration.

**Supplementary Information:**

The online version contains supplementary material available at 10.1007/s10508-023-02661-z.

## Introduction

Sexual orientation refers to one’s relatively enduring sexual attraction to men, women, both, or neither (i.e., asexuality). Individuals exclusively sexually attracted to the same sex are the minority (< 5% in Western countries), however, about 10% of men and women self-identify as non-heterosexual (e.g., “homosexual,” “gay,” “lesbian,” “bisexual,” etc.), and 17% of men and 34% of women report to be moderately to predominantly sexually attracted to the same sex (Bailey et al., 2016; Rahman et al., 2020). Men and women differ greatly in several aspects of their sexual orientation. In women, bisexuality is more prominent than in men, while in men an exclusive same-sex sexual orientation is more common. Women are also reported to be more fluid in their sexual orientation across the life-span, and their sexual orientation is discussed to be more socially and situationally influenced than that of men (Diamond, 2007a, 2007b, 2016). Importantly, up-to-date research strongly argues in favor of differing biological underpinnings of male and female sexual orientation (Bogaert & Skorska, 2020; Breedlove, 2017).

Aggression has been defined as behavior intended to harm another individual, for example due to feelings of anger in response to frustration (hostile aggression; Berkowitz, 1993). While several evolutionary theories suggest a link between same-sex sexual behavior, increased affiliation and reduced aggression (Kirkpatrick, 2000; Muscarella, 2000; Rahman & Wilson, 2003), it has recently been suggested that same-sex sexual attraction evolved as one of several adaptive social traits promoting prosocial behavior (Barron & Hare, 2020). These traits responded to the selection for increased social integration and reduced aggression during the evolutionary process of self-domestication (natural selection for prosocial behavior; Hare, 2017). Due to the social function of sexual behavior (e.g., reinforcing social bonds, appeasement, pacification, stress reduction), some degree of same-sex sexual attraction, as a motivator for same-sex sexual behavior, would have been a selective advantage, strengthening within-sex social bonds and reducing intragroup conflict (Barron & Hare, 2020). In fact, gay men have been shown to describe themselves as less aggressive than heterosexual men (Dickins & Sergeant, 2008; Ellis et al., 1990; Gladue & Bailey, 1995; Sergeant et al., 2006), more empathetic, and more agreeable than heterosexual men (Lippa, 2005, 2008; Salais & Fischer, 1995; Sergeant et al., 2006). They also show stronger neural activation during empathizing (Perry et al., 2013). Since (heterosexual) men are considered to be more aggressive than (heterosexual) women (Archer, 2004), the reduced aggressiveness reported by gay men can be interpreted as reflecting a “cross-sex shift.” Atypical exposure to prenatal androgens is discussed as one possible mechanism underlying this cross-sex shift (for review and discussion, see Bogaert & Skorska, 2020; Breedlove, 2017). Since sex differences in aggression are discussed to be related to prenatal androgen exposure (Hines, 2020), organizational effects of androgen hormones provide a proximate link between sexual orientation and aggression (Gladue & Bailey, 1995).

Corresponding data on aggression in women are scarce, and some reports of reduced as well as heightened physical aggression in lesbians compared to heterosexual women have to be regarded cautiously, due to fairly small sample sizes and quite liberal criteria for determining same-sex sexual preferences (Ellis et al., 1990; Gladue, 1991; for discussion, see Gladue & Bailey, 1995). Gladue and Bailey (1995), however, found no significant differences between lesbians’ and heterosexual women’s self-reported physical aggression. Similarly, no meaningful differences between lesbians and heterosexual women regarding agreeableness could be shown (Lippa, 2005, 2008).

To our knowledge, there is no study that actually examined how sexual orientation might affect the perception of social aggression cues, thus elucidating potential mechanisms underlying sexual orientation related differences in aggression. Here, a series of experiments is presented, investigating the effects of sexual orientation on the neural processing of chemosensory aggression signals (Studies 1a and 1b) and visual aggression signals (Studies 2a and 2b). Chemosensory aggression signals, present within axillary sweat, were chosen because such signals are considered to act as inherently “honest signals” (for discussion, see Lübke et al., 2017); hence, their effects should be valid universally (i.e., they are unaffected by cultural or social learning factors). Moreover, sexual orientation has already been shown to affect responses to non-emotional axillary sweat (Lübke et al., 2012; Martins et al., 2005), as well as to sweat compounds probably involved in the communication of aggression (Lübke & Pause, 2014; Lübke et al., 2009). It has repeatedly been shown that chemosensory signals are processed as highly relevant, and exert significant behavioral effects without being consciously perceived as odors (de Groot et al., 2015; Pause et al., 2010, 2020). Such weakly salient social signals typically reveal robust between-sex differences in social perception (Lee et al., 2017; Li et al., 2008; Pause et al., 2010, 2020), hence they should be the most potent type of signal to reveal effects of sexual orientation as well. Visual aggression signals, i.e., angry faces, were chosen as the perception of non-emotional human faces has already been shown to vary with sexual orientation (Ishai, 2007; Kranz & Ishai, 2006; Rahman & Yusuf, 2015; Steffens et al., 2013). Because sexual orientation affects empathy-related processes (Lübke et al., 2020; Nettle, 2007; Perry et al., 2013; Ruben et al., 2014), sexual orientation-related effects on the processing of facial affect were expected. In order to match the chemosensory signals, and to enhance ecological validity, weak facial expressions were presented.

Gay men’s reduced aggressiveness and their heightened empathic traits suggest fine-tuned socioemotional sensitivity and effective social information processing. It was therefore expected that gay men would show stronger preferential processing of aggression signals than heterosexual men, indicative of heightened socioemotional sensitivity. Since research so far does not support sexual orientation related differences in aggression in women, and because sexual orientation is considered to be more fluid in women than in men, it was here expected that lesbian and heterosexual women would not differ in their processing of aggression signals. Thus, based on theoretical and design-related (contradicting hypotheses regarding the effects of male and female sexual orientation) considerations, and second, because running separate studies avoids redundant group comparisons and thereby increases statistical power in the separate designs, men and women were examined in individual studies (men: Studies 1a and 2a, women: Studies 1b and 2b). Any comparison of study results was then based on confidence intervals rather than significance tests (in accordance with Nieuwenhuis et al., 2011).

## Study 1a: Men’s Neural Responses to Chemosensory Aggression Signals

### Introduction

Social communication is one of the main functions of human chemosensation (Pause, 2012; Stevenson, 2010). Stress, fear, and anxiety (for overviews, see de Groot & Smeets, 2017; Lübke & Pause, 2015; Pause, 2017), as well as happiness (de Groot et al., 2015) and disgust (Zheng et al., 2018) have been shown to be chemosensorily communicated via axillary sweat. Such emotional chemosignals exert emotion-specific behavioral effects (e.g., fear chemosignals facilitating the detection of fearful but not other negative facial expressions, Kamiloğlu et al., 2018), demonstrating their high ecological validity (also see de Groot et al., 2014, 2015; Prehn et al., 2006; Zheng et al., 2018). Reliability has been examined by the first meta-analysis on the chemosensory communication of anxiety, fear and stress (de Groot & Smeets, 2017), revealing a small-to-moderate effect size (Hedges’ *g*: 0.36) and evidence for a true effect (*p*_s_ < 0.0001). A growing body of literature now shows that aggression is communicated in a similar fashion: Aggression related chemosignals heighten physiological arousal (Adolph et al., 2010), shift attention toward anxiety relevant information (Mutic et al., 2016), and modulate limbic system activation (Mutic et al., 2017). Most recent results show a reliable and differential brain response to chemosensory aggression signals in men, as indicated by chemosensory event-related potentials (CSERP). Men process female aggression signals as more significant than female control sweat (enlarged P3-2 amplitudes, Pause et al., 2020).

Study 1a examines the effects of male sexual orientation on the neural processing of chemosensory aggression signals by means of the highly time-sensitive ERP technique, expanded by source localization using current source density (CSD) and low resolution electromagnetic tomography analyses (LORETAs). Indicators of both early, pre-attentive stimulus processing (P2 peak) as well as late, evaluative stimulus processing (P3-1 and P3-2 peaks) were assessed. While the chemosensory P2 peak reflects the processing of specific stimulus features and automatic attention allocation, both the P3-1 and the P3-2 are sensitive to the subjective stimulus significance (Lübke et al., 2012; Pause et al., 1996). The amplitudes of these components reflect the strength of the neural response, while the latencies are indicative of the respective processing speed. CSD analyses and LORETA reveal the cortical sources of the neural energy involved in the different processing stages with the same high temporal resolution as the EEG (in contrast to the much slower functional magnetic resonance imaging; fMRI).

An interaction of sexual orientation and type of chemosensory signal on the strength of early and late processing (P2, P3-1 and P3-2 amplitude) was hypothesized: Indicating heightened socioemotional sensitivity, gay men were expected to show stronger processing (i.e., larger amplitudes) of chemosensory aggression signals vs. non-emotion control sweat, and, most importantly, to show stronger processing of chemosensory aggression signals than heterosexual men. The neural sources of these effects were explored by LORETA and CSD analyses. Further, gay men’s processing speed (P2, P3-1, and P3-2 latencies) of aggression signals vs. non-emotional control sweat was expected to differ, and their processing speed of aggression signals should differ from that of heterosexual men. Since both accelerated (Lübke et al., 2017) and delayed (Pause et al., 2020) neural responses may relate to preferential stimulus processing, no specific direction of this effect could be predicted.

### Method

#### Participants

Participants were recruited via advertisement at the university and in social networks. From originally 74 men interested, 45 men could be included into the current study. However, data of another two men had to be excluded because they did not succeed in correctly performing the required breathing technique (see Stimulus Presentation), data of one man were excluded due to pronounced EEG artefacts (see EEG data reduction), and data of two other men due to technical problems with the olfactometer, resulting in final sample of 40 male participants. These participants either reported being heterosexual or disclosed as being gay (or “homosexual”) upon being asked an open question about their sexual orientation (“How do you define your sexual orientation?”). In total, 17 men disclosed as gay and 23 men reported being heterosexual. The heterosexual men were the same as in Pause et al. (2020) and, accordingly, the data are the same. Both heterosexual and gay men were recruited simultaneously. At the time of their participation, none of them reported any bisexual sexual behavior (as assessed via the Kinsey Scale, ranging from 0 = *exclusively heterosexual* to 6 = *exclusively homosexual*, Kinsey et al., 1948). Gay men described both their sexual behavior (*M* = 5.9, *SD* = 0.3) and their sexual fantasies (*M* = 5.8, *SD* = 0.4) as “exclusively homosexual,” while heterosexual men described both as “exclusively heterosexual” (behavior: *M* = 0.0, *SD* = 0.0, fantasies: *M* = 0.0, *SD* = 0.2). Further exploration of the participants’ sexual orientation revealed that all heterosexual men disagreed to the statement “I can imagine being in a love relationship with a man,” while all gay men agreed. The participants in total had a mean age of 25.4 years (*SD* = 5.4, range = 20–43), and age did not differ with sexual orientation (*p* = 0.112). Further, they had to be of excellent physical and mental health, meeting an array of inclusion criteria of not reporting a history of chronic medication or the use of drugs, not smoking cigarettes on a regular basis, and not suffering from any neurological, psychiatric, endocrine or immunological condition. No participant suffered from diseases related to the upper respiratory system or had had any nasal surgery. All participants were required to be of European origin, minimizing any effects of culture or ethnos on chemosensory perception. Moreover, all participants were right-handed (as assessed by means of the Annett Handedness Questionnaire; Annett, 1970). None of the participants had acted as a sweat donor for the current study. A brief olfactory screening test revealed no suspicion of general hyposmia in any participant (see Pause et al., 2020). Due to not meeting one or more of these inclusion criteria, 29 applicants were not included into the current study.

#### Chemosensory Stimuli

##### Sweat Sampling Procedure

Detailed information regarding the methods and the results of the sweat donation are presented in Pause et al. (2020). In brief, axillary sweat was obtained via cotton pads fixed in the armpits of 17 heterosexual women and 17 heterosexual men. Only heterosexual individuals were included in order to ensure sufficient statistical power (i.e., to avoid the need for inclusion of an additional factor in later analyses), and because heterosexual individuals’ sweat is of higher ecological validity, since encounters with heterosexual individuals are the majority in daily life. The sweat donors attended two sessions on separate days. During the first session, hostile aggression was induced (aggression condition) via frustration, while the second session served as a non-emotional control condition. In the aggression session, the donors were exposed to the Point Subtraction Aggression Paradigm (PSAP; Carré & McCormick, 2008). Here, the donors’ task was collecting as many points as possible via button presses, while a fictitious opponent simultaneously stole those points (frustration). The donors then could choose between three behavioral strategies, one of which was related to overt aggressive behavior against their opponent by repeatedly hitting a specific button in order to erase a certain number of points from their opponent’s account. These points were not added to the donors account, but completely withdrawn, hence the donors did not gain anything else from this behavior. The points they collected, on the other hand, were later exchanged for money. In the control session, the PSAP was replaced by a construction computer game.

As was to be expected in response to frustration, almost all donors (30 of 34) showed overt hostile aggressive behavior during the PSAP by hitting the respective button during the game (*M* = 17.2% of the intra-individual behavior, *SD* = 13.8%). At the beginning of each session and after the PSAP and the construction game, respectively, the donors reported on their feelings via six visual analogue scales (anger, disgust, fear, happiness, sadness, surprise; 0 = “*not at all*” to 10 = “*extremely*”). They reported a stronger increase of anger during the aggression session compared to the control session (*p* < 0.001), an emotional response typically involved in hostile aggression. Moreover, the donors reported a more prominent decrease of fear during the aggression compared to the control session (*p* = 0.046), while the other emotions were not differentially affected. Physiologically, the donors showed an increase of their salivary testosterone level during the aggression session (*p* = 0.045). Their mean baseline-corrected heartrate decreased during the control session (*p* = 0.001), but did not change during the aggression session.

Following the completion of collection, all cotton pads were chopped and pooled with respect to the donor’s sex and the donation condition. Each of the final four homogenized samples (male aggression, male control, female aggression, female control) was divided into 100 portions of 0.4 g cotton pad and stored at − 20 °C.

##### Stimulus Presentation

For EEG recording and stimulus ratings, the chemosensory stimuli were presented according to the method described by Kobal and Hummel (1988), using a constant-flow (100 ml/s; stimulus duration = 0.4 s) 8-channel olfactometer (OL023, Burghart, Wedel, Germany). Both nostrils were stimulated simultaneously (for details see supplementary material, Part A).

#### Odor Detection, Odor Ratings, and Emotional Ratings

Following each stimulus presentation during the EEG recording (see below), participants indicated whether they had perceived an odor (yes, no), and afterwards, independently of their detection statement, their opinion on whether the putative odor sample had been obtained from women or men. Participants indicated either answer by ticking a box on the screen (yes/ no or male/ female) with the mouse (forced choice). In order to not bias the participants and to ensure attention, participants were told that odors would only be presented in some, but not all trials. In fact, however, odors were presented in all trials and no blank trials were included. Hit rates were calculated, defined as the percentage of correct answers in relation to the total number of trials (detection rate, e.g., indicating “yes” in each of the 25 presentations of male aggression signals would result in a detection rate of male aggression signals of 100%). Missing data in the detection task were treated as “not detected.”

Odor ratings were obtained prior to EEG recording. Each sample was presented via the olfactometer once for 0.4 s for each of three ratings. The order of odor presentation was randomized. Participants rated the sweat samples’ intensity on a pictographic scale ranging from 1 (“*not at all*”) to 9 (“*extremely*”). In addition, participants selected terms from a list of 147 verbal descriptors which, according to their opinion, best described the sweat samples’ odorous quality (Dravnieks et al., 1984). Here, participants were required to select at least one descriptor, but were free to select as many descriptors as they deemed fitting. Participants practiced using the descriptor list for as long as they needed to by describing the odor of phenyl–ethyl alcohol, which was used in the hyposmia screening.

In order to judge the donors’ affect during sweat donation, participants reported to what extent they thought the donors had felt fear, anger, and happiness on visual analogue scales (0 = “*not at all*” to 10 = “*extremely*”).

#### EEG Procedure

The procedure of the entire session is presented in Figure S1. During EEG recording, 100 stimuli were presented, with 25 presentations of each sweat sample (male aggression, male control, female aggression, female control). The stimuli were presented in a previously randomized, fixed order. Participants were informed that they would receive body odors; however, they did neither know anything about the emotional state of the odor donors, nor how many different odors they would receive. EEG recordings were subdivided into 3 blocks (33, 33, and 34 trials) separated by two individually adjusted resting periods. On average, the EEG procedure lasted 42 min (*SD* = 5 min). For details on the trial structure, see supplementary material, part A.

#### Data Recording and Reduction

Details of data recording and reduction are presented in the supplementary material, part A. In brief, ongoing EEG was recorded from 61 scalp locations. For later correction of ocular artefacts, an additional electrode was placed 1.5 cm below the right eye, outside the vertical pupil axis, to record vertical eye movements. Fp2 was used to record the horizontal eye movements. The ground electrode was placed at position FT10. Data were sampled at 500 Hz with an averaged reference, and low-pass filtered online at 135 Hz using a QuickAmp 72 EEG amplifier (Brain Products GmbH, Gilching, Germany).

Offline, EEG signals were re-referenced to linked ear lobes, filtered, corrected for eye movements (Gratton et al., 1983) and baseline-corrected (-500 ms – 0 ms before stimulus onset). Afterwards, channels containing artefacts (i.e., voltage bursts) were rejected, and any trials having more than 1/3 of channels in one or more pools (see below) contaminated with artefacts were excluded from analysis. Data of four participants were consequently excluded from the study due to less than 13 of 25 trials remaining in at least one condition.

For peak detection, signals were low pass filtered with 7 Hz, 48 dB/octave. The 61 scalp electrode positions were subdivided into nine areas (pools) and a mean peak for each pool was calculated by averaging adjacent electrodes in anterior (a), central (c), and posterior (p) areas for the left (l) and the right (r) hemisphere as well as for midline (m) electrodes (see supplementary material, part A). In relation to the baseline period, three separate peaks were detected in predefined latency windows in each pool (P2: 500–700 ms, P3-1: 700–900 ms, P3-2: 900–1100; Pause & Krauel, 2000), and amplitudes and latencies of each peak were calculated (also see Pause et al., 2020).

#### Data Analyses

Detection rates, ratings of intensity, and the attribution of the donors’ sex were analyzed by means of three-way mixed-factors ANOVAs, including the within-subjects factors Emotion (EMO; aggression sweat sample, control sweat sample), Donors’ Sex (DS; male sweat sample, female sweat sample), and the between-subjects factor Participants’ Sexual Orientation (SO; heterosexual, same-sex oriented). Detection rates for each sweat sample (male aggression, male control, female aggression, female control) were additionally tested against chance level by means of one-sample *t*-tests. In order to investigate whether participants could identify the emotional content of the sweat samples, the suspected emotions of the donors were analyzed by means of a two-way mixed-factors ANOVA separately for each sweat sample, including the within-subjects factor Assessed Emotion (AE: anger, fear, happiness) and the between-subjects factor SO. Any effects only evident in heterosexual individuals will be reported for the sake of completeness but not discussed, since they have already been discussed in Pause et al. (2020). ERP analyses were based on the full sample of *n* = 40 men. Due to technical errors, some rating data were only available from *n* = 39 (intensity ratings), *n* = 38 (suspected affective state of female aggression sweat donors and male and female control sweat donors), and *n* = 37 men (suspected affective state of male aggression sweat donors). Preliminary EEG data analysis, reported in detail in the supplementary material, showed that each peak was most prominent within posterior electrode pools (as compared to anterior or central pools, see Tables S1 and S2 in the supplementary material, part B). Thus, in order to reduce noise and increase statistical power, peaks detected within these pools were averaged and subjected to further analysis. Amplitudes and latencies of the CSERP components were subjected to three-way mixed-factors ANOVAs, including the within-subjects factors EMO and DS, and the between-subjects factor SO. Significant interactions were followed up by nested effects analysis (Page et al., 2003). In all analyses, the alpha level was set to* p* < 0.05 (based on Huynh–Feldt corrected degrees of freedom).

Differences between gay and heterosexual men’ processing of aggression signals were further explored by CSD analyses and LORETAs. CSD maps were calculated using a spherical spline model (Perrin et al., 1989, order of splines: m = 4, maximal degree of Legendre polynominals = 20). LORETA was used in order to localize the source of brain activity (Pascual-Marqui et al., 1994). The source space comprises 2394 voxel at 7 mm spatial resolution, covering the cortical gray matter and the hippocampus (Pascual-Marqui et al., 1999), defined via a reference brain from the Brain Imaging Center at the Montreal Neurological Institute (Collins et al., 1994). LORETA uses a 3-shell spherical head model, co-registered to the Talairach anatomical brain atlas (Talairach & Tournoux, 1988).

### Results

#### Stimulus Detection and Assessment of Donors’ Sex

During EEG recording, men (*n* = 40) detected on average 52.78% (*SD* = 26.45%) of the presented sweat samples, not differing from chance in their overall detection performance (*p* = 0.511) or when detection performance was analyzed individually for each sweat sample (all *p*_*s*_ ≥ 0.097). However, male sweat was detected more often than female sweat (DS: *F*(1, 38) = 12.04, *p* = 0.001, *η*^*2*^_*p*_ = 0.24, Power = 0.92). Neither men’s sexual orientation nor the emotional content of the sweat samples did affect the detection performance (all *p*_*s*_ ≥ 0.184).

Men’s assessment of the donors’ sex did not differ from chance (detection rate = 51.35%, *SD* = 6.19%; *p* = 0.176). The same was true when assessment performance was analyzed separately for male and female aggression and control sweat (all *p*_*s*_ ≥ 0.215). Again, neither men’s sexual orientation nor the emotional content of the sweat samples did affect the detection performance (all *p*_*s*_ ≥ 0.078). All group mean values regarding stimulus detection (Table S3) and donors’ sex assessment (Table S4) are presented in the supplementary material, part C.

#### Odor Ratings and Descriptions

##### Intensity

Men (*n* = 39) judged the sweat samples’ intensity overall as relatively weak (*M* = 3.19, *SD* = 1.39), and rated male sweat samples’ intensity slightly higher (*M* = 3.54, *SD* = 1.74) than female sweat samples’ (*M* = 2.83, *SD* = 1.41; DS: *F*(1, 37) = 8.53, *p* = 0.006, *η*^*2*^_*p*_ = 0.19, Power = 0.81). However, intensity ratings were unaffected by men’s sexual orientation and the emotional content of the sweat samples (all *p*_*s*_ ≥ 0.205; for all group mean values see Table S5, supplementary material, part C).

##### Suspicion of the Donors’ Affective State

In general, any emotion the male participants suspected the sweat donors to have experienced during sweat donations was rated as very low in intensity (*n* = 39, *M* = 1.85, *SD* = 1.27). Judgements of either sweat sample did not differ with respect to men’s sexual orientation or the assessed emotion, indicating that the participants could not consciously recognize anger or aggression from the sweat samples (all *p*_*s*_ ≥ 0.080; for all group mean values see Table S6 in the supplementary material, part C).

##### Verbal Descriptors

Out of the 147 verbal descriptors the participants could choose from, they selected the descriptors “medicinal,” “warm,” and “sweaty” most often to describe male aggression sweat. “Light” was chosen most often and “warm” was chosen second most often to describe the other sweat samples, (together with “stale” in case of female control sweat; for the frequency distribution of the selected verbal descriptors see Figures S2 and S3 in the supplementary material, part C).

#### Chemosensory Event-Related Potentials

##### Amplitudes

Mean differences and 95% confidence intervals for each effect are presented in the supplementary material (Table S7, part C). Early processing of chemosensory signals (P2) in men is affected by their sexual orientation. Irrespective of the particular chemosensory signal presented, gay men generally display larger P2 amplitudes than heterosexual men (SO: *F*(1, 38) = 6.58, *p* = 0.014, *η*^*2*^_*p*_ = 0.15, Power = 0.70). Moreover, men respond with larger P2 amplitudes to male compared to female sweat samples (DS: *F*(1, 38) = 4.12,* p* = 0.049, *η*^*2*^_*p*_ = 0.10, Power = 0.51).

**Fig. 1:**
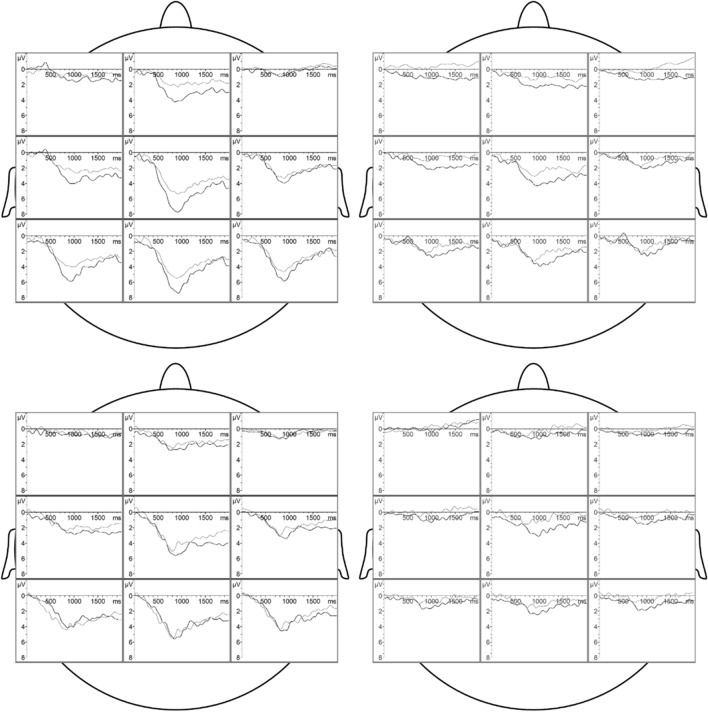
Grand averages of the chemosensory event-related potentials (CSERPs) across gay men (left column) and heterosexual men (right column) in response to male (upper row) and female (lower row) sweat. Black lines indicate CSERPs to aggression sweat, and grey lines indicate CSERPs to control sweat. Time point 0 refers to the valve activation

Men’s P3-1 amplitude is differentially affected by sexual orientation, and both the donors’ emotion and their sex: As hypothesized, gay men showed larger P3-1 amplitudes than heterosexual men when presented with male aggression sweat (SO x EMO x DS: *F*(1, 38) = 5.57, *p* = 0.024, *η*^*2*^_*p*_ = 0.13, Power = 0.63; nested effects: SO in male sweat in aggression sweat: *F*(1, 38) = 8.87, *p* = 0.005, *η*^*2*^_*p*_ = 0.19, Power = 0.83; restricting the main effect SO to responses to male aggression sweat, see Table 1, Fig. 1). In line, gay men respond with larger P3-1 amplitudes to male aggression sweat compared to male control sweat (based on the same SO x EMO x DS interaction; nested effects: EMO in male sweat in gay men: *F*(1, 38) = 9.43, *p* = 0.004, *η*^*2*^_*p*_ = 0.20, Power = 0.85). Moreover, gay men also showed larger P3-1 amplitudes when presented with male compared to female aggression sweat (based on the same SO x EMO x DS interaction; nested effects: DS in aggression sweat in gay men: *F*(1, 38) = 8.02, *p* = 0.007, *η*^*2*^_*p*_ = 0.17, Power = 0.79, restricting the main effect DS to gay men’s responses to aggression sweat, see Table 1).

**Table 1:** Overall analysis of variance of the amplitudes of the chemosensory event-related potentials (CSERPs) in men: Significant main effects, interactions, and single comparisons

Effect	P2 amplitude	P3-1 amplitude	P3-2 amplitude
SO	GM > HM*	GM > HM*	GM > HM*
DS	MS > FS*	MS > FS*	MS > FS*
SO x EMO x DS		GM > HM in MS in AS** AS > CS in MS in GM** MS > FS in AS in GM**	
EMO			AS > CS*

SO, Sexual Orientation; GM, gay men, HM, heterosexual men, EMO, Emotion; AS, anger sweat, CS, control sweat, DS, Donors’ Sex; MS, male sweat, FS, female sweat

*p ≤ .05. **p ≤ .01

Similar to the P2 amplitude, irrespective of the particular chemosensory signal presented, gay men display larger P3-2 amplitudes than heterosexual men (SO: *F*(1, 38) = 6.69, *p* = 0.014, *η*^*2*^_*p*_ = 0.15, Power = 0.71). Moreover, men respond with larger P3-2 amplitudes to male compared to female sweat samples (DS: *F*(1, 38) = 5.75, *p* = 0.022, *η*^*2*^_*p*_ = 0.13, Power = 0.64). Finally, men’s P3-2 amplitudes in response to aggression sweat are larger than amplitudes in response to control sweat (EMO: *F*(1, 38) = 6.42, *p* = 0.016, *η*^*2*^_*p*_ = 0.15, Power = 0.69). The distribution of CSERPs across the scalp is depicted in Fig. 1.

##### Latencies

Mean differences and 95% confidence intervals for each effect are presented in the supplementary material (Table S8, part C). Men’s P2 peak appears with a longer latency in response to male compared to female sweat (DS: *F*(1, 38) = 9.70, *p* = 0.003, *η*^*2*^_*p*_ = 0.20, Power = 0.86). While the latency of the P3-1 peak is unaffected by any experimental condition (all *p*_*s*_ ≥ 0.285), men’s P3-2 latency is longer upon presentation of female compared to male control sweat (EMO x DS: *F*(1, 38) = 4.48, *p* = 0.041, *η*^*2*^_*p*_ = 0.11, Power = 0.54; nested effects: DS in control sweat: *F*(1, 38) = 5.50,* p* = 0.024, *η*^*2*^_*p*_ = 0.13, Power = 0.63, see Table 2).

**Table 2:** Overall analysis of variance of the latencies of the chemosensory event-related potentials (CSERPs) in men: Significant main effects, interactions, and single comparisons

Effect	P2 latency	P3-1 latency	P3-2 latency
DS	MS > FS**		
EMO x DS			FS > MS in CS*

EMO, Emotion; AS, anger sweat; CS, control sweat; DS, Donors’ Sex; MS, male sweat; FS, female sweat

*p ≤ .05. **p ≤ .01

#### Current Source Densities

**Fig. 2:**
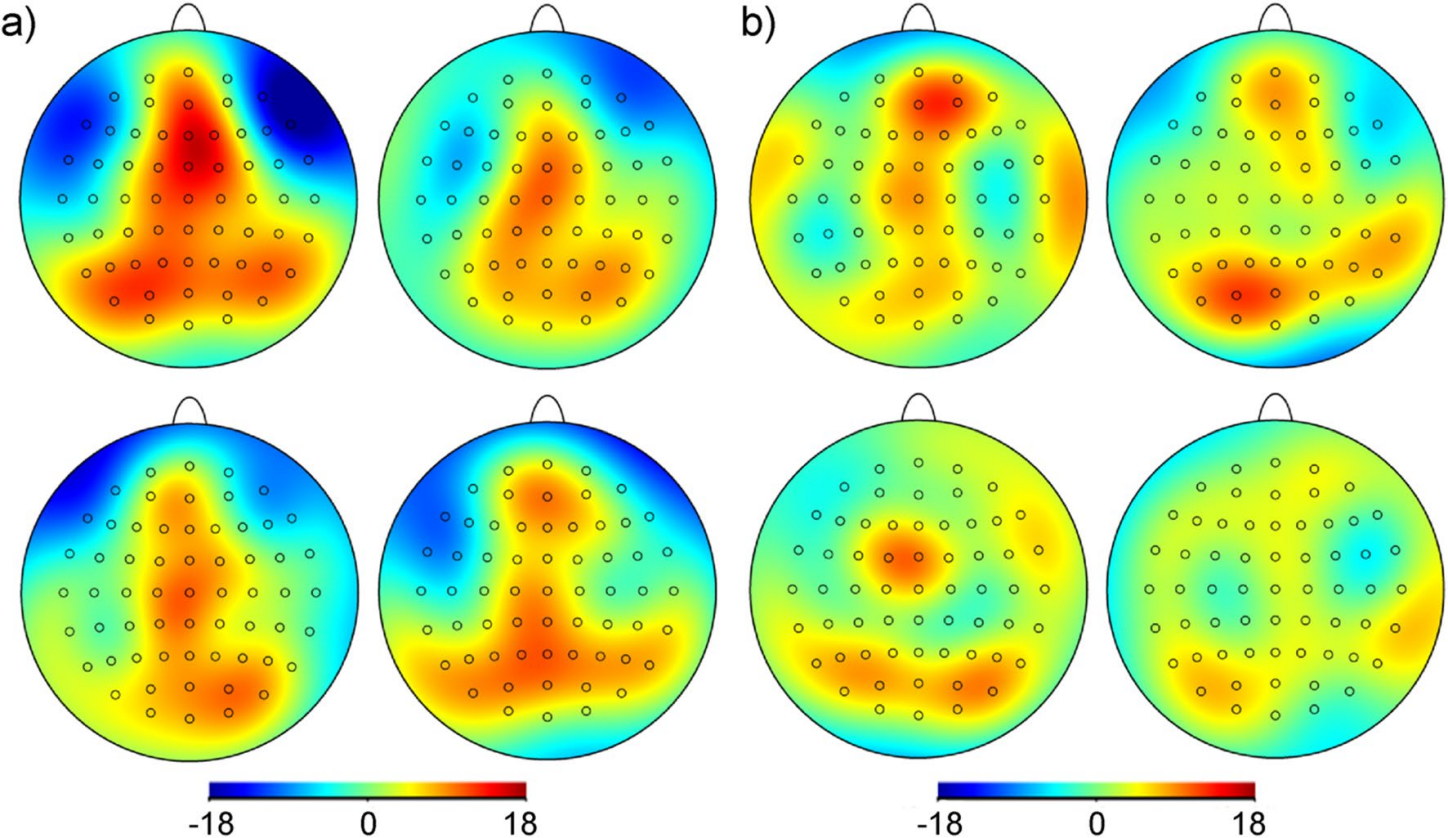
Current source density (CSD, µV/m^2^) maps (two-dimensional smoothing for a view across all electrodes) at the time of the total mean P3-1 peak latency (804 ms). Panel **a** CSD maps of gay men in response to male aggression sweat (upper left), male control sweat (upper right), female aggression sweat (lower left), and female control sweat (lower right). Panel **b** CSD maps of heterosexual men in response to male aggression sweat (upper left), male control sweat (upper right), female aggression sweat (lower left), and female control sweat (lower right). Red colors represent cortical activation (neuronal sources), and blue colors represent cortical deactivation (neuronal sinks)

CSD analyses focused on the P3-1 latency range, since gay and heterosexual men showed the most prominent differences in the strength of the P3-1 following presentation of (male) aggression sweat: Within this time-frame, gay men’s neocortical responses to male aggression sweat appeared particularly pronounced (see Fig. 2a): Prominent cortical sources largely spread from frontopolar to parietal areas along the midline, as well as across the entire posterior scalp region, there extending to cover both lateral areas. Simultaneously, inhibition was prominent bilaterally across fronto-temporal areas. When presented with male control sweat, gay men responded with a similar distribution of cortical sinks and sources, however, both activation and deactivation were less intense, and neural activation was less widespread. In response to female sweat samples, gay men’s activation was also prominent along the midline, extending to lateral parieto-occipital areas, however, it was less pronounced than in response to male aggression sweat.

Heterosexual men responded to male aggression sweat with cortical activation along the midline, ranging from frontopolar to occipital sites, but the activation was much weaker than in gay men (see Fig. 2b). Moreover, a marked concurrent inhibition in heterosexual men was absent; instead, lateral areas were activated simultaneously. In response to male control sweat, left sided parieto-occipital activation was dominant. In response to female aggression sweat, parietal areas were bilaterally activated. Neural responses to female control sweat appeared extremely weak and disperse.

#### Low Resolution Electromagnetic Tomography Analyses

**Fig. 3:**
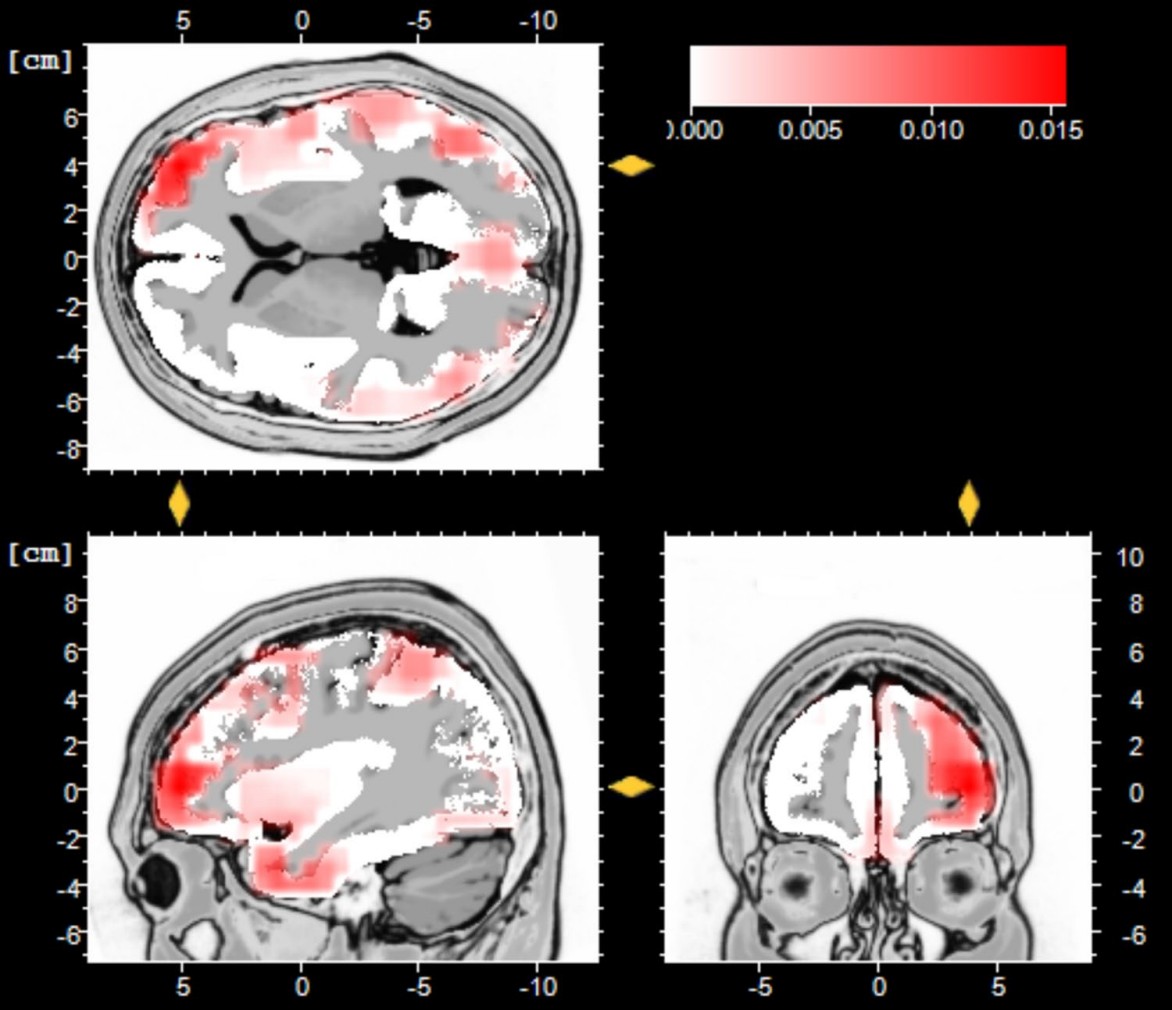
Low resolution electromagnetic tomography analysis (LORETA) maps depicting the location of the maximum current density (in µA/mm^2^) at the time of the total mean P3-1 latency (804 ms) of gay men’s in contrast to heterosexual men’s responses to male aggression sweat

Using the same rationale as with the CSD analyses, LORETA compared gay and heterosexual men’s responses to male aggression sweat within the P3-1 latency range: When contrasting gay men’s responses to male aggression sweat with that of heterosexual men, peak activation appeared within right anterior prefrontal cortex (inferior frontal gyrus; IFG, BA 10, see Fig. 3).

### Discussion

The current study is the first to show that men’s sexual orientation affects the neural processing of chemosensory aggression signals, which might indeed reflect heightened sensitivity towards social aggression signals and highly effective social information processing in gay men. As the chemosensory stimuli were hardly recognized as odors, and because sexual orientation did not affect the detection performance or any rating measure, these brain responses are unlikely to be mediated consciously.

Gay compared to heterosexual men respond with enhanced neural energy to all kinds of chemosignals utilized here, both during early, pre-attentive (P2 amplitude, prominent above posterior scalp regions see Lübke et al., 2012) and late, evaluative processing stages (P3-2 amplitude, prominent above posterior scalp regions, see Polich, 2007). This pattern implies that chemosensory social signals in general, irrespective of the specific information they transmit, are processed as subjectively highly relevant by gay men (Krauel et al., 1998a; Lübke et al., 2017; Pause et al., 2010) and automatically capture their attentional resources (Krauel et al., 1998b).

As expected, gay men showed larger P3-1 amplitudes (prominent above posterior scalp regions; see Polich, 2007) in response to male aggression sweat compared to male control sweat, and also compared to female aggression sweat. Further, their P3-1 amplitude in response to male aggression sweat is more pronounced than that of heterosexual men. These findings are in line with a processing advantage for chemosensory aggression signals in gay men (Pause et al., 2020). The respective cortical sources were located at frontopolar to parietal areas along the midline, as well as across the whole posterior scalp region, with concurrent inhibition of fronto-lateral regions (CSD maps). This might reflect activation of the mirror neuron system, indicative of a general contagious effect of social emotions (Hoenen et al., 2018), along with an inhibition of cortical regions involved in higher order reasoning, such as executive functions (dorsolateral prefrontal cortex; Gilbert & Burgess, 2008). Occipitotemporal sources might relate to activation of the fusiform gyrus, possibly coding the “human” quality within the chemosensory signal (Prehn-Kristensen et al., 2009). Temporo-parietal regions are involved in Theory of Mind processes (Schurz et al., 2014), and might thus relate to a heightened capacity or predisposition towards attributing the mental state of others (here inferred from chemosensory signals) in gay men. Contrasting gay men’s responses to male aggression sweat with that of heterosexual men revealed peak activation in the right inferior frontal gyrus (IFG, BA 10, LORETA). The IFG is considered to be part of a response inhibition network (Puiu et al., 2020), also involved in the incidental regulation of negative emotions (Payer et al., 2012). In contrast, women have been shown to activate dorsomedial prefrontal areas in response to male chemosensory aggression signals, in line with an immediate response selection (Pause et al., 2020).

Thus, although the current results indicate that gay men respond highly sensitive to chemosensory to chemosensory aggression signals, they relate to reduced aggression in gay men nevertheless (Dickins & Sergeant, 2008; Ellis et al., 1990; Gladue & Bailey, 1995; Sergeant et al., 2006), mediated by effective spontaneous emotion regulation. These findings thus are in line with the idea, that same-sex sexual attraction evolved together with prosocial traits and hence, reduced aggression, in order to ease social integration (Barron & Hare, 2020). Same-sex sexual attraction and reduced aggression should have a high probability of co-occurring when they have evolved under the same selective pressure, and the current results might mirror such co-occurrence (but see Barron, 2021 for discussion).

## Study 1b: Women’s Neural Responses to Chemosensory Aggression Signals

### Introduction

Study 1a revealed that sexual orientation affects neural responses to chemosensory aggression signals in men. While both theoretical and empirical reports had predicted this effect, in comparison, empirical data on the relationship between female sexual orientation and aggression are by far scarcer, and rather inconclusive: Reports range from heightened aggression (Ellis et al., 1990) to reduced aggression (Gladue, 1991) and to null results (Gladue & Bailey, 1995) when comparing lesbian and heterosexual women. The contradicting results probably reflect the heightened diversity in lesbian women regarding self-perception and self-labelling, as well as the relatively high sexual fluidity in women (Diamond, 2007b, 2016). Then again, even evolutionary theories disagree on the direction of a possible link between aggression and female sexual orientation: For example, sensu Barron and Hare (2020) same-sex sexual attraction and reduced aggression would have been beneficial for males and females alike. Rahman and Wilson (2003), on the other hand, suggest that female masculinization should have been beneficial throughout evolution, allowing for same-sex sexual behavior aiding in alliance formation with powerful females, and heightened aggression supporting offspring protection.

Study 1b was designed in order to investigate possible effects of female sexual orientation on the neural processing of chemosensory anxiety signals, following the same protocol as Study 1a. In contrast to study 1a, it was here expected that lesbian and heterosexual women would not differ in their processing of chemosensory aggression signals. Most current results demonstrate enhanced (P3-1 and P3-2 amplitudes) and prolonged (P3-1 latency) evaluative processing of especially male chemosensory aggression signals in women (Pause et al., 2020). It was expected that such differential neural processing of chemosensory aggression signals would occur irrespective of women’s sexual orientation.

### Method

#### Participants

Women were recruited via the same channels as men in Study 1a. From 109 women interested in participating, 46 women could be included into the current study; however, data of two women had to be excluded from CSERP analysis due to pronounced EEG artefacts (see EEG data reduction), resulting in final sample of 44 female participants. These women either reported being heterosexual (*n* = 25) or disclosed as being lesbian (or “homosexual”; *n* = 19) upon being asked an open question about their sexual orientation (“How do you define your sexual orientation?”). The heterosexual women were the same as in Pause et al. (2020), and accordingly, the data are the same. Both heterosexual and lesbian women were recruited simultaneously. At the time of participation, none of them reported any bisexual sexual behavior (as assessed via the Kinsey Scale, ranging from 0 = *exclusively heterosexual* to 6 = *exclusively homosexual*; Kinsey et al., 1948). On average, lesbian women described their sexual behavior (*M* = 5.7, *SD* = 0.5) as “exclusively homosexual”, but their sexual fantasies as “predominantly homosexual, only incidentally heterosexual” (*M* = 5.1, *SD* = 0.8). Heterosexual women described their sexual behavior as well as their sexual fantasies as “exclusively heterosexual” (behavior: *M* = 0.1, *SD* = 0.4, fantasies: *M* = 0.4, *SD* = 0.6). Further exploration of their sexual orientation revealed that only 79% of the lesbian women disagreed to the statement “I can imagine being in a love relationship with a man,” but 21% stated “neither, nor”. Of the heterosexual women, 96% agreed, but 4% also stated “neither, nor.” The participating women in total had a mean age of 24.7 years (*SD* = 3.9, range = 19–35), and age did not differ with sexual orientation (*p* = 0.700). They all met the same inclusion criteria as the men from Study 1a, and additionally had a regular menstrual cycle and did not use hormonal contraceptives. Based on reports of the regular cycle length and the actual cycle day (defining the 5 midcycle days as “ovulatory phase”), lesbian and heterosexual women did not differ in their respective cycle phase at the time of their participation (follicular phase: *n* = 22, ovulatory phase: *n* = 9, luteal phase: *n* = 13; *p* = 0.274, Fisher’s Exact test). None of the participating women had acted as a sweat donor for the current study. Due to not meeting one or more of these inclusion criteria, another 63 applicants had been excluded from the current study.

#### Chemosensory Stimuli; Odor Detection, Odor Ratings, and Emotional Ratings; EEG Procedure

The methodological details were identical to Study 1a.

#### Data Recording and Reduction

Data recording and reduction were carried out the same way as in Study 1a. Here, data of two women were completely excluded from analyses due to less than 13 of 25 trials free of artefacts remaining in at least one condition.

#### Data Analyses

Data analyses were identical to those in Study 1a, except that here the levels of the factor Sexual Orientation (SO) are labelled “lesbian” and “heterosexual,” respectively. ERP analyses were based on the full sample of 44 women. Due to technical errors, some rating data were only available from 43 (intensity ratings, suspected affective state of male and female aggression sweat donors) and 42 women (suspected affective state of female control sweat donors). Similar to Study 1a, preliminary EEG data analysis, reported in detail in the supplementary material, showed that each peak was most prominent within posterior electrode pools (as compared to anterior or central pools, see Tables S9 and S10 in the supplementary material, part D); thus, peaks detected within these pools were averaged and subjected to further analysis. In case lesbian and heterosexual women would, conforming to the hypotheses, not differ significantly in the strength of their neural responses to aggression signals, no CSD analyses or LORETAs were performed.

### Results

#### Stimulus Detection and Assessment of Donors’ Sex

Women (*n* = 44) detected on average 51.39% (*SD* = 24.81%) of the sweat samples, the overall detection performance not differing from chance (*p* = 0.713). However, the detection performance regarding male aggression sweat exceeded chance level (*M* = 63.09%, *SD* = 27.18%, *t*(43) = 3.195, *p* = 0.003, *d* = 0.48), while the detection of none other sweat sample differed from chance (all *p*_*s*_ ≥ 0.190). Moreover, women detected both male and female aggression sweat samples more often than male and female control sweat samples, respectively (EMO x DS: *F*(1, 42) = 8.52, *p* = 0.006, *η*^*2*^_*p*_ = 0.17, Power = 0.81; nested effects: EMO in male sweat: *F*(1, 42) = 19.88, *p* < 0.001, *η*^*2*^_*p*_ = 0.32, Power = 0.99; EMO in female sweat: *F*(1, 42) = 4.37, *p* = 0.043, *η*^*2*^_*p*_ = 0.09, Power = 0.53; confirming the main effect EMO: *F*(1, 42) = 19.45, *p* < 0.001, *η*^*2*^_*p*_ = 0.32, Power = 0.99). Women further detected male aggression sweat more often than female aggression sweat (based on the same interaction EMO x DS; nested effects: DS in aggression sweat: *F*(1, 42) = 16.75, *p* < 0.001, *η*^*2*^_*p*_ = 0.29, Power = 0.98; restricting the main effect DS: *F*(1, 42) = 17.12, *p* < 0.001, *η*^*2*^_*p*_ = 0.29, Power = 0.98 to aggression sweat samples only). Women’s sexual orientation did not affect the sweat samples’ detection (*p* ≥ 0.208).

Women correctly assigned the donors’ sex to 52.55% (*SD* = 5.33) overall, exceeding chance level (*t*(43) = 3.17, *p* = 0.003, *d* = 0.48). Especially male aggression sweat was correctly assigned to the male sex more frequently than chance (*t*(43) = 3.33, *p* = 0.002, *d* = 0.50). The sex assignment of the other sweat samples did not differ from chance (all *p*_*s*_ 0.614). Accordingly, women correctly assigned male aggression sweat to the male sex more often than female aggression sweat to the female sex (EMO x DS: *F*(1, 42) = 4.10, *p* = 0.049, *η*^*2*^_*p*_ = 0.09, Power = 0.51; nested effects: DS in aggression sweat: *F*(1, 42) = 5.64, *p* = 0.022, *η*^*2*^_*p*_ = 0.12, Power = 0.64), and male aggression sweat more often than male control sweat (based on the same interaction EMO x DS; nested effects: EMO in male sweat: *F*(1, 42) = 5.63, *p* = 0.022, *η*^*2*^_*p*_ = 0.12, Power = 0.64). Women’s sexual orientation did not affect sex assignment performance (all *p*_*s*_ ≥ 0.092). All group mean values regarding stimulus detection (Table S11) and donors’ sex assessment (Table S12) are presented in the supplementary material, part E.

#### Odor Ratings and Descriptions

##### Intensity

Women (*n* = 43) rated the sweat samples’ intensity overall as relatively weak (*M* = 3.03, *SD* = 1.53), but provided slightly higher intensity ratings for aggression (*M* = 3.30, *SD* = 2.05) compared to control sweat samples (*M* = 2.76, *SD* = 1.30; EMO: *F*(1, 41) = 7.17, *p* = 0.011, *η*^*2*^_*p*_ = 0.15, Power = 0.74). Women’s sexual orientation did not affect intensity ratings (all *p*_*s*_ ≥ 0.065; for all group mean values see Table S13, supplementary material, part E).

##### Suspicion of the Donors’ Affective State

Women rated any emotion the sweat donors might have experienced during sweat donations as rather weak in intensity (*n* = 44, *M* = 2.18, *SD* = 1.46). In detail, women falsely suspected the donors of male aggression sweat to have experienced more anxiety than anger or happiness (*n* = 43; AE: *F*(2, 82) = 7.59, *p* = 0.001, *η*^*2*^_*p*_ = 0.16, Power = 0.94; anger vs. anxiety: *t*(42) = 2.54, *p* = 0.015, *d* = 0.39; anxiety vs. happiness: *t*(42) = 3.47, *p* = 0.001, *d* = 0.53). Regarding male control sweat, lesbian women (*M* = 3.44, *SD* = 2.65) even assigned higher ratings of anxiety to the donors’ emotion during sweat donation than heterosexual women (*M* = 1.76, *SD* = 16.93; *n* = 39; SO x AE: *F*(2, 74) = 3.22, *p* ≤ 0.050, *η*^*2*^_*p*_ = 0.08, Power = 0.60; nested effects: SO in anxiety: *F*(1, 37) = 4.58, *p* = 0.039, *η*^*2*^_*p*_ = 0.11, Power = 0.55). Regarding female aggression sweat, women correctly suspected its donors to have experienced more anger than happiness during donation (*n* = 43; AE: *F*(2, 82) = 4.09, *p* = 0.029, *η*^*2*^_*p*_ = 0.09, Power = 0.71; anger vs. happiness: *t*(42) = 2.567, *p* = 0.014, *d* = 0.39). The donors of female control sweat, on the other hand, were suspected to have experienced more happiness than anxiety (*n* = 42; AE (*F*(2, 80) = 3.90,* p* = 0.037, *η*^*2*^_*p*_ = 0.09, Power = 0.69; anxiety vs. happiness: *t*(41) = 2.226, *p* = 0.032, *d* = 0.34; for all group mean values see Table S14 in the supplementary material, part E).

##### Verbal Descriptors

Women chose “Light” most often, and “warm” second most often for describing each of the four sweat samples (for the frequency distribution of the selected verbal descriptors see Figures S4 and S5 in the supplementary material, part E).

#### Chemosensory Event-Related Potentials

##### Amplitudes

Mean differences and 95% confidence intervals for each effect are presented in the supplementary material (Table S15, part E). As expected, women’s sexual orientation did not affect the amplitudes of the P2, P3-1, and P3-2 components following the presentation of aggression sweat (all *p*_*s*_ ≥ 0.141). Instead, lesbian women display larger P2 amplitudes in response to male control sweat than heterosexual women (SO x EMO x DS: *F*(1, 42) = 5.99, *p* = 0.019, *η*^*2*^_*p*_ = 0.13, Power = 0.66; nested effects: SO in male sweat in control sweat: *F*(1, 42) = 6.44, *p* = 0.015, *η*^*2*^_*p*_ = 0.13, Power = 0.70). Correspondingly, lesbian women showed larger P2 amplitudes in response to male compared to female control sweat (based on the same interaction SO x EMO x DS; nested effects: DS in lesbian women in control sweat: *F*(1, 42) = 5.30, *p* = 0.026, *η*^*2*^_*p*_ = 0.11, Power = 0.61). Only heterosexual women showed larger P2 amplitudes in response to male aggression sweat compared to male control sweat (based on the same interaction SO x EMO x DS; nested effects: EMO in heterosexual women in male sweat: *F*(1, 42) = 4.16, *p* = 0.048, *η*^*2*^_*p*_ = 0.09, Power = 0.51).

The P3-1 amplitude, however, is larger in response to aggression sweat compared to control sweat in women irrespective of their sexual orientation (EMO: *F*(1, 42) = 4.37, *p* = 0.043, *η*^*2*^_*p*_ = 0.09, Power = 0.53). The P3-1 is further affected by the sweat donors’ sex, as is the P3-2, both with larger amplitudes in response to male compared to female sweat (P3-1, DS: *F*(1, 42) = 10.44, *p* = 0.002, *η*^*2*^_*p*_ = 0.20, Power = 0.88; P3-2, DS: *F*(1, 42) = 12.66, *p* = 0.001, *η*^*2*^_*p*_ = 0.23, Power = 0.94).

**Fig. 4:**
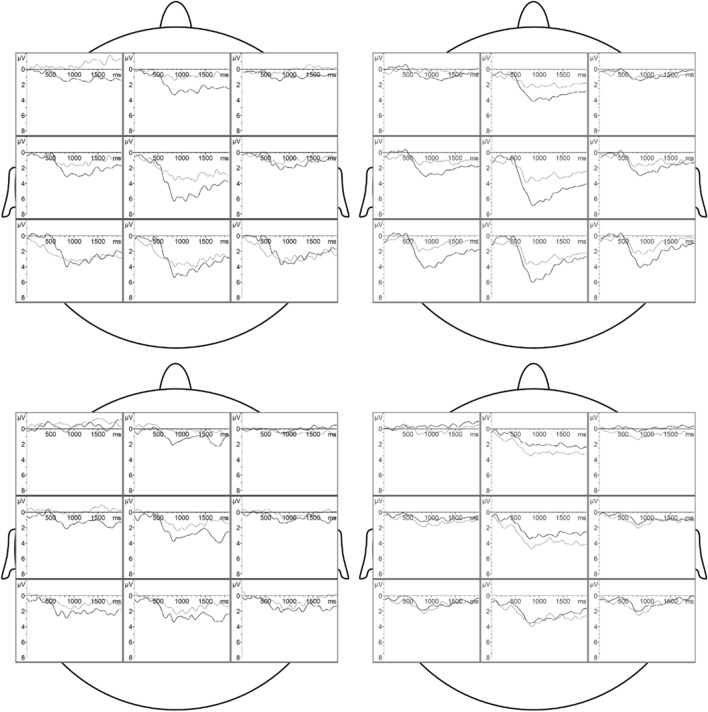
Grand averages of the chemosensory event-related potentials (CSERPs) across lesbian women (left column) and heterosexual women (right column) in response to male (upper row) and female (lower row) sweat. Black lines indicate CSERPs to aggression sweat, and grey lines indicate CSERPs to control sweat. Time point 0 refers to the valve activation

For a comprehensive overview of the significant ANOVA results, see Table 3; the distribution of CERPs across the scalp is depicted in Fig. 4.

**Table 3:** Overall analysis of variance of the amplitudes of the chemosensory event-related potentials (CSERPs) in women: Significant main effects, interactions, and single comparisons

Effect	P2 amplitude	P3-1 amplitude	P3-2 amplitude
DS		MS > FS**	MS > FS***
SO x EMO x DS	LW > HW in MS in CS* AS > CS in MS in HW* MS > FS in CS in LW*		
EMO		AS > CS*	

SO, Sexual Orientation; LW, lesbian women; HW, heterosexual women; EMO, Emotion; AS, anger sweat; CS, control sweat; DS, Donors’ Sex; MS, male sweat; FS, female sweat

*p ≤ .05. **p ≤ .01. ***p ≤ .001

##### Latencies

Mean differences and 95% confidence intervals for each effect are presented in the supplementary material (Table S16, part E). Similar to the amplitudes and according to the hypotheses, women’s sexual orientation did not affect the latencies of the P2, P3-1, and P3-2 components following the presentation of aggression sweat (all *p*_*s*_ ≥ 0.078).

Women’s P2 latencies were generally unaffected by any experimental condition (all *p*_*s*_ ≥ 0.085, except for an interaction SO x DS: *F*(1, 42) = 4.40, *p* = 0.042, *η*^*2*^_*p*_ = 0.10, Power = 0.53 not yielding significant effects within the nested effects analyses, see Table 4).

**Table 4:** Overall analysis of variance of the latencies of the chemosensory event-related potentials (CSERPs) in women: Significant main effects, interactions, and single comparisons

Effect	P2 latency	P3-1 latency	P3-2 latency
SO x DS	(*)		
SO x EMO		LW > HW in CS* AS > CS in HW*	

SO, Sexual Orientation; LW, lesbian women; HW, heterosexual women; EMO, Emotion; AS, anger sweat; CS, control sweat; DS, Donors’ Sex; MS, male sweat; FS, female sweat

*p ≤ .05. (*) = interaction significant, but nested effects analyses not significant

Women’s P3-1 latencies, on the other hand, were differentially affected: In response to control sweat, lesbian women showed longer latencies of the P3-1 than heterosexual women ( SO x EMO: *F*(1, 42) = 5.48, *p* = 0.024, *η*^*2*^_*p*_ = 0.12, Power = 0.63; nested effects: SO in control sweat: *F*(1, 42) = 4.91, *p* = 0.032, *η*^*2*^_*p*_ = 0.11, Power = 0.58). Heterosexual women display longer latencies of the P3-1 in response to aggression compared to control sweat (based on the same interaction SO x EMO; nested effects: EMO in heterosexual women: *F*(1, 42) = 4.62, *p* = 0.037, *η*^*2*^_*p*_ = 0.10, Power = 0.55).

The latencies of the P3-2 peak again were, like those of the P2, unaffected by the experimental conditions (all *p*_*s*_ ≥ 0.078).

#### Current Source Densities and Low Resolution Electromagnetic Tomography Analyses

Since lesbians and heterosexual women did not differ in the strength of their neural responses to aggression sweat, it was refrained from conducting CSD analyses and LORETAs.

### Discussion

The results of Study 1b showed that, as expected, women generally display enhanced evaluative processing of aggression sweat compared to control sweat (P3-1 amplitude), the effect not depending on their sexual orientation. This effect most probably relates to the inherent social significance of the chemical aggression signal (Pause et al., 2020), but might additionally have been driven by detecting an odor from the aggression sweat samples (as indicated by heighetend intensity ratings and detection rates; for discussion, see Pause et al., 2020). The significance lies in male chemosensory aggression signals indicating the presence of an aggressive man, increasing the likelihood of dangerous encounters especially for physically inferior women. Contrasting with the results from Study 1a, these findings suggest that underlying neurodevelopmental factors might differ in men and women. Gladue and Bailey (1995) suggested that, in men, sexual orientation and aggression-related “traits” are shaped simultaneously by common underlying neurohormonal factors during brain development, while these developmental processes appear to diverge in women.

Interestingly, female sexual orientation comes into play during the processing of sex-related chemosensory information. Lesbian women respond with larger P2 amplitudes to male control sweat than heterosexual women, and also to male compared to female control sweat, but without, consciously identifying the sweat donors’ sex. An enlarged P2 reflects the chemosensory signal’s capacity to automatically catch attentional neural resources due to their inherent significance (Krauel et al., 1998b), indicating that male sweat appears highly relevant to lesbian women. This might relate to lesbians’ reduced exposure to male sweat due to fewer close encounters with men compared to heterosexual women (and compared to close encounters with other women, see Diamond & Dubé, 2002). A lesbian’s brain might thus interpret a male chemosignal as significant in a sense of “unusual” or “new.” In total, women’s brains appear to focus aggression-related information, and lesbian women’s brains additionally respond to sex-related information.

## Study 2a: Men’s Neural Responses to Visual Aggression Signals

### Introduction

Study 1a showed that male sexual orientation affects neural responses to subtle chemosensory aggression signals favoring gay men, and Study 2a was designed in order to test whether similar effects occur in response to subtle visual aggression signals (i.e., angry faces).

So far, research has focused on sex-related rather than on sexual orientation-related differences in the processing of emotional visual stimuli, and prominent sex differences have been shown repeatedly: Women outperform men in recognizing subtle emotional facial expressions (Hoffmann et al., 2010), and show stronger and more differential pre-attentive processing of subtle negative facial expressions (Lee et al., 2017; Xu et al., 2013). Importantly, women usually display pronounced processing of especially negative (e.g., angry faces) and weakly salient (e.g., expressions of weak intensity) emotional stimuli from early to late processing stages (Li et al., 2008; Lithari et al., 2010). fMRI and behavioral data show that sexual orientation affects the processing of male and female faces (Ishai, 2007; Kranz & Ishai, 2006; Rahman & Yusuf, 2015; Steffens et al., 2013), somewhat unrelated to facial affect (see Rahman & Yusuf, 2015 for discussion). However, to date no study has directly evaluated neural responses to facial affect with respect to sex and sexual orientation, although research indicates that sexual orientation affects empathy-related processing (Lübke et al., 2020; Nettle, 2007; Perry et al., 2013; Ruben et al., 2014), supporting possible sexual orientation-related effects on the processing of facial affect.

For Study 2a, pictures of angry faces were morphed with neutral faces of the same model to create an emotional intensity of 20%, and were presented for 500 ms only, in order to result in visual social signals comparable to chemosensory aggression signals in their subtlety. Since sex differences range from early to late processing stages, both the face-sensitive N170 component (also varying with facial affect, see Hinojosa et al., 2015) and the late, evaluative P3 component were examined. In line with study 1a, gay men were expected to show stronger processing (larger N170 and P3 amplitudes) of subtle angry vs. neutral faces, and, most importantly, to show stronger processing of angry faces than heterosexual men, indicative of heightened emotional sensitivity. Exploratorily, the speed of early and late processing (N170 and P3 latencies) was inspected for effects of sexual orientation and type of visual signal.

### Method

#### Participants

The data of Study 2a were obtained from the same sample of initially 45 men as in Study 1a. Here, data of one man had to be excluded from analysis due to technical issues during EEG recording, resulting in a total of 44 men. Thus, the sample comprised of 39 whose data were included for analyses in Study 1a, plus 5 additional participants. Study 2a was conducted simultaneously to Study 1a. Of these, 21 men disclosed as gay, and 23 men reported being heterosexual. Participants had a mean age of 24.9 years (*SD* = 5.3, range = 18–43), and age did not differ with sexual orientation (*p* = 0.095). To ensure that all participants had adequate eyesight, visual acuity was assessed using Landolt rings (EN ISO 8596, Oculus GmbH, Wetzlar, Germany) and was always better than 70% normal vision.

#### Visual Stimuli

Participants were presented with pictures of faces of 10 Caucasian models (5 women), displaying both neutral and angry expressions (Radboud Faces Database; Langner et al., 2010), resulting in a total of 20 different pictures. Angry expressions were morphed with the respective neutral faces in order to achieve an intensity of 20 percent for the anger expressions (FaceMorpher 2.51 Multi, Luxand Inc., Alexandria, VA, see Figure S6 in the supplementary material, part F). All pictures were scaled at 409 × 614 pixels.

#### Emotional Ratings

In order to investigate how the participants would judge the intensity of the facial affect they inferred from the pictures, participants rated to what extent they thought each models’ face displayed fear, anger, and happiness on visual analogue scales (0 = “*not at all*” to 10 = “*extremely*”). These ratings were obtained following each stimulus presentation during the EEG recording.

#### EEG Procedure

During EEG recording, 80 stimuli were presented, with 20 presentations of each class of stimuli (male anger face, male neutral face, female anger face, female neutral face). Each of the individual pictures was presented four times. The stimuli were presented in a previously randomized, fixed order. EEG recordings were subdivided into 2 blocks (á 40 trials), separated by a resting period, the duration of which was individually adjusted to each participant. On average, the EEG part, including the break, lasted 28 min (*SD* = 2 min). For details of the EEG procedure, see supplementary material, part F.

#### Data Recording and Reduction

EEG recording and data reduction were identical to Studies 1a and 1b (see supplementary material, part A), with the exception that here, data were filtered using a low pass filter of 20 Hz (48 dB/octave) and a high pass filter of 0.1 Hz (48 dB/octave). In relation to the baseline period, two separate peaks were differentiated within predefined latency windows (N170: 130–220 ms, P3: 280–480 ms). These peaks were detected within posterior electrode pools due to their prominent posterior dominance (Bentin et al., 1996; Polich, 2007; Rousselet et al., 2004), averaged across the transversal line, and amplitudes and latencies of each peak were calculated.

#### Data Analysis

Amplitudes and latencies of the CSERP components were subjected to three-way mixed-factors ANOVAs, including the within-subjects factors Emotion (EMO; angry face, neutral face), and Faces’ Sex (FS; male face, female face), and the between-subjects factor Sexual Orientation (SO; same-sex oriented, heterosexual). Significant interactions were followed up by nested effects analysis (Page et al., 2003). In all analyses, the alpha level was set to *p* < 0.05 (based on Huynh–Feldt corrected degrees of freedom). In order to investigate whether participants could identify the respective facial expressions, the suspected emotions were analyzed by means of a two-way mixed-factors ANOVA separately for each class of stimuli (angry male face, neutral male face, angry female face, neutral female face) including the within-subjects factor Assessed Emotion (AE: anger, fear, happiness) and the between-subjects factor SO. Here, data of 1 participant had to be excluded due to him having misunderstood the respective instructions (*n* = 43 men). Since amplitudes of neither component varied with sexual orientation (see results), it was refrained from performing CSD analyses and LORETAs, respectively.

### Results

#### Emotional Ratings

In general, men judged the intensity of any emotion present within the facial expressions as rather weak (*M* = 1.40, *SD* = 0.98). They rated angry and neutral faces of either sex as predominantly angry, but also more fearful than happy (AE: all *p*_s_ < 0.001). Sexual orientation did not affect the emotional ratings (all *p*_s_ ≥ 0.489). A comprehensive overview of the ANOVA results and descriptive values are presented in Tables S17 and S18 in the supplementary material, part G.

#### Amplitudes of the Visual Event-Related Potentials

In men, the amplitudes of the N170 (all *p*_*s*_ ≥ 0.359) as well as the amplitudes of the P3 (all *p*_*s*_ ≥ 0.056) were unaffected by any experimental condition. VERPs across the scalp, separated for the experimental conditions are depicted in Figure S7 in the supplementary material, part G.

#### Latencies of the Visual Event-Related Potentials

**Fig. 5:**
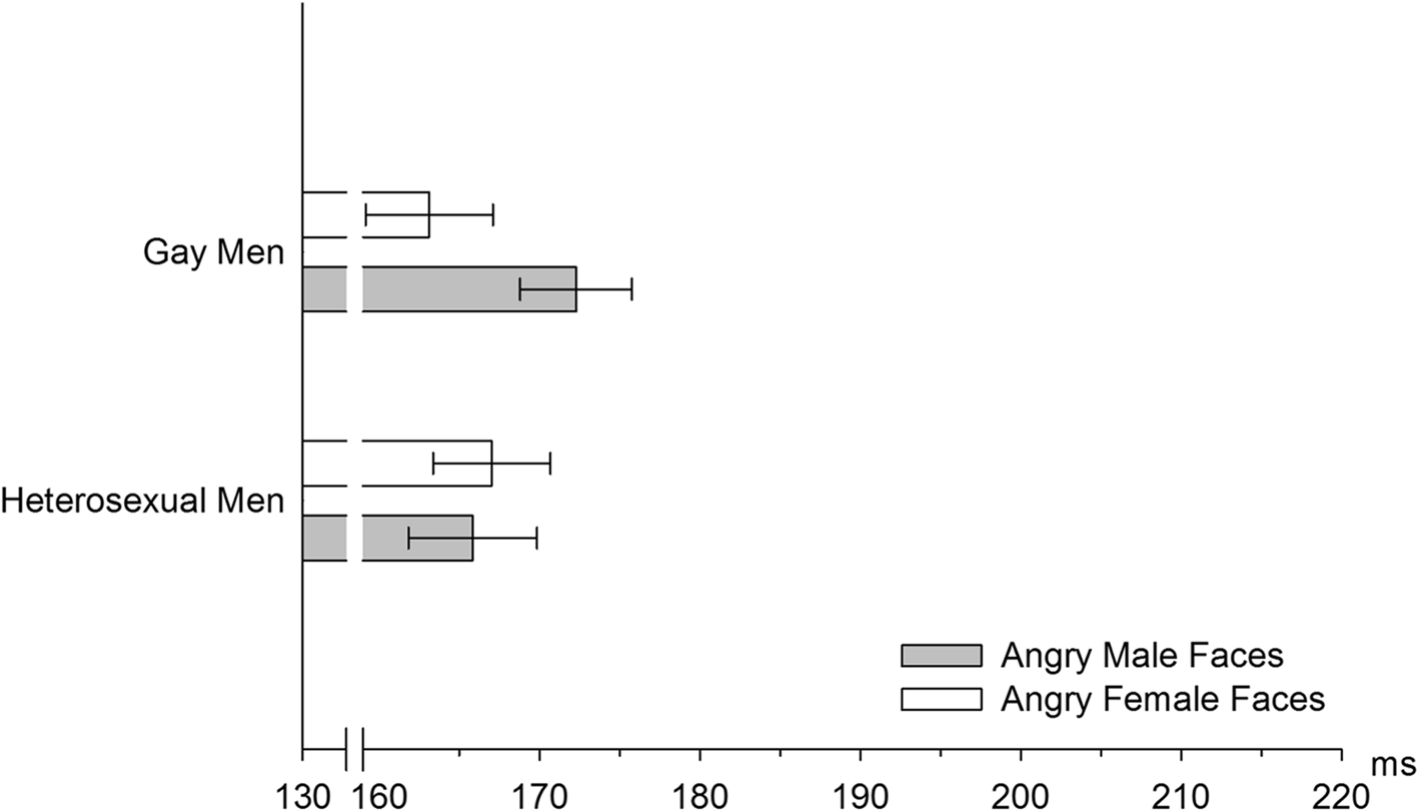
Men’s mean latencies (± *SEM*) of the N170 component upon presentation of angry male and angry female faces

Gay men showed longer latencies of the N170 peak in response to angry male as compared to angry female faces (SO x EMO x FS: *F*(1, 42) = 5.72, *p* = 0.021, *η*^*2*^_*p*_ = 0.12, Power = 0.65; nested effects: FS in angry faces in gay men: *F*(1, 42) = 6.24, *p* = 0.016, *η*^*2*^_*p*_ = 0.13, Power = 0.68, see Fig. 5, see Table S19 for mean difference and confidence interval).

### Discussion

Study 2a showed that male sexual orientation does not directly affect the processing of subtle angry faces, but that gay men respond with longer latencies of the face specific N170 to angry male compared to angry female faces. Overall, neither early nor late processing was affected by the type of facial expression.

Prolonged latencies of the N170 in response to negative facial affect, as evident in gay men in response to male vs. female angry faces, have been discussed as being related to the activation of subcortical pathways upon exposure to social signals of danger, the information of which needing time for integration (Batty & Taylor, 2003). Typically, the amplitude of the N170 is regarded as indicating preferential processing of the most relevant social signal (i.e., an angry face signaling danger; Blau et al., 2007; Caharel et al., 2005; Hinojosa et al., 2015), and larger N170 amplitudes to angry male than angry female faces have been reported (Valdes-Conroy et al., 2014). The current data thus might indicate that gay men process the relative degree of danger signaled by angry human faces at an early processing stage, probably reflecting heightened socioemotional sensitivity.

The facial expressions were generally judged as relatively weak in intensity, and neutral and angry faces alike were rated as predominantly slightly angry. On one hand, these ratings indicate that the current methods (morphing and short presentation time) were successful in creating truly subtle visual aggression signals. On the other hand, they give a hint as to why neither the N170 nor the P3 differed with respect to facial affect. Neutral faces might not be processed as simply neutral, but ambiguous (Cooney et al., 2006; Hagemann et al., 2016; Marusak et al., 2017; Tottenham et al., 2013), and are, in fact, commonly judged as negative ("negativity bias", Cooney et al., 2006; Hagemann et al., 2016; Marusak et al., 2017; Tottenham et al., 2013). In any case, sexual orientation did not affect the ratings, indicating that gay men’s suggested advantage in differential processing of visual social aggression signals is not related to perceived signal unambiguity.

## Study 2b: Women’s Neural Responses to Visual Aggression Signals

### Introduction

Study 1b already revealed that lesbian and heterosexual women did not differ in their processing of chemosensory aggression signals. Studies examining possible effects of female sexual orientation on social perception and processing in the visual domain are similarly scarce. From the few studies, some showed differences between lesbian and heterosexual women (Ishai, 2007; Kranz & Ishai, 2006; Rahman & Yusuf, 2015; Steffens et al., 2013), and others do not (Steffens et al., 2013). These contradicting findings might be related to the heightened diversity in lesbian women regarding self-perception and self-labelling, as well as their sexual fluidity (Diamond, 2007a, 2007b, 2016).

Study 2b was designed in order to examine possible effects of female sexual orientation on the neural processing of weakly salient facial expressions of anger, posed by men and women. The experimental protocol was the same as in Study 2a. It was here expected that lesbian and heterosexual women would not differ in their processing of visual aggression signals.

### Method

#### Participants

The data of Study 2b were obtained from the same pool of initially 46 women as in Study 1b. Here, data of one woman had to be excluded from analysis due to pronounced EEG artefacts (see EEG data reduction), resulting in a total of 45 women. Thus, the sample comprised 43 women whose data were included for analyses Study 1b, plus two additional participants. Study 2b was conducted simultaneously to Study 1b. Of these, 19 women disclosed as being lesbian, and 26 women reported being heterosexual. Participants had a mean age of 24.8 years (*SD* = 3.9, range = 18–43), and age did not differ with sexual orientation (*p* = 0.997), neither did menstrual cycle phase (follicular phase: *n* = 22, ovulatory phase: *n* = 10, luteal phase: *n* = 13; *p* = 0.173, Fisher’s Exact test).

#### Visual Stimuli; Emotional Ratings; EEG Procedure

The methodological details were identical to study 2a.

#### Data Recording and Reduction

EEG recording and data reduction were identical to Study 2a. Here, data of one woman were excluded from analyses due to less than 10 of 20 trials free of artefacts remaining in at least one condition.

#### Data Analyses

Data analyses were identical to those in Study 2a, except that here the levels of the factor Sexual Orientation (SO) were labelled “lesbian” and “heterosexual,” respectively. Since amplitudes of neither component varied with sexual orientation (see results), it was refrained from performing CSD analyses and LORETAs.

### Results

#### Emotional Ratings

Women rated the intensity of any emotion present within the facial expressions as weak (*M* = 1.59, *SD* = 1.13). They rated angry and neutral faces of either sex as predominantly angry, but also more fearful than happy. Only neutral male faces were rated similarly angry and fearful but still more so than happy (AE: all *p*_*s*_ < 0.001). As with men, emotional ratings were not affected by sexual orientation (all *p*_*s*_ ≥ 0.334). A comprehensive overview of the ANOVA results and descriptive values are presented in Tables S21 and S22 in the supplementary material, part H.

#### Amplitudes of the Visual Event-Related Potentials

Women’s N170 (all *p*_*s*_ ≥ 0.144; except for an interaction EMO x FS: *F*(1, 43) = 5.55, *p* = 0.023, *η*^*2*^_*p*_ = 0.11, Power = 0.63 not yielding significant effects within the nested effects analyses) and P3 amplitudes (all *p*_*s*_ ≥ 0.102) were not affected by any experimental condition. VERPs across the scalp, separated for the experimental conditions are depicted in Figure S8 in the supplementary material, part H.

#### Latencies of the Visual Event-Related Potentials

In women, the latencies of the N170 (all *p*_*s*_ ≥ 0.422) as well as the latencies of the P3 (all *p*_*s*_ ≥ 0.072) were not affected by any experimental condition.

### Discussion

In line with the hypotheses, Study 2b showed no evidence that women’s sexual orientation would affect the processing of subtle anger expressions. These results relate to previous research showing that lesbian and heterosexual women similarly do not differ in their self-reported aggression (Gladue & Bailey, 1995) or in social perception (Steffens et al., 2013). Contrasting with the results from Study 2a, these findings again hint at the hypothesized underlying neurodevelopmental factors differing between men and women (Bogaert & Skorska, 2020; Breedlove, 2017; Gladue & Bailey, 1995, see Study 1b).

Similar to men, women’s neural responses did not differ with respect to facial affect (see Study 2a), and women, like men, inferred anger as the most prominent emotion not only from the angry, but also from the neutral expressions, questioning the “neutrality” of the neutral faces similar to the results of Study 2a (Cooney et al., 2006; Hagemann et al., 2016; Marusak et al., 2017; Tottenham et al., 2013). Obviously, women processed both the neutral and the weak anger expressions as negatively valenced, which relates to no apparent differences in their neural responses.

## General Discussion

In a series of four studies, it could be shown that male but not female sexual orientation affects the processing of aggression-relevant social signals. These results shed light on the general sociobehavioral relevance of sexual orientation, beyond the context of sexual partner preferences.

So far, data had shown that gay men report a reduced propensity towards aggression (Dickins & Sergeant, 2008; Ellis et al., 1990; Gladue, 1991; Gladue & Bailey, 1995; Sergeant et al., 2006), and self-describe as more empathetic and more agreeable than heterosexual men (Lippa, 2005, 2008; Salais & Fischer, 1995; Sergeant et al., 2006). The current results now support and expand these results by demonstrating sexual orientation related differences in basic social perception in men. Gay men appear highly sensitive to the significance of subtle chemical social signals of aggression (P3-1 amplitude), while predominantly processing these signals in brain areas involved in response inhibition and spontaneous regulation of negative emotions (IFG; Payer et al., 2012; Puiu et al., 2020). Gay men thus might be equipped to recognize aggressive tendencies of another (male) individual early on, and might be able to respond with flexible behavior, e.g., appeasement and de-escalation instead of fight, due to emotion regulation and response inhibition. This pattern of adaptive social information processing links male same-sex sexual orientation to reduced aggression, a link which is proposed in classic and contemporary adaptive evolutionary theories on same-sex sexual orientation in humans (Barron & Hare, 2020; Kirkpatrick, 2000). These theories focus on social integration (or “alliance formation”; see Kirkpatrick, 2000) as the selective driving force, having promoted traits which aided in social integration, such as heightened prosociality and reduced aggression on one hand, and same-sex sexual behavior or same-sex sexual attraction on the other hand. Thus, the current results, showing that in men, a same-sex oriented sexual orientation as a trait is linked to reduced aggression, might reflect a co-occurrence of traits which co-evolved under the same selective pressure.

The current studies further revealed substantial differences in how social information processing is affected by sexual orientation in men and women, especially in the chemosensory domain (for confidence intervals of the mean differences see Tables S7, S8 in part C, S15, S16 in part E, S19 in part G, and S22 in part H in the supplementary material). As expected, the effect that gay compared to heterosexual men process human chemosignals with more neural energy (P2, P3-1, and P3-2 amplitudes) had no equivalent in women. Moreover, while gay men and lesbian women display pronounced differential processing of aggression sweat (P3-1 amplitude), gay men differ from heterosexual men while lesbians do not differ from heterosexual women (see Tables S7 and S15). In line, recent meta-analyses on cognitive abilities have revealed that typically gay men perform quite differently from heterosexual men, while lesbian and heterosexual women perform quite similar (Xu et al., 2017, 2020). Differences between lesbians’ and heterosexual women’s cognitive abilities only emerge in tasks typically favoring men, such as mental rotation (Xu et al., 2017). Because socioemotional processing, as examined in the current study, is commonly regarded a “women’s domain,” the fact that here lesbians and heterosexual women did not differ fits with the referenced research. The sex-depending domain specific effects of sexual orientation might be explained by differences in the neurobiological basis of sexual orientation in men and women (Bogaert & Skorska, 2020; Breedlove, 2017). Biological factors promoting female same-sex sexual orientation possibly shift the brain to only subtly differ from heterosexual women’s in a given domain, while factors promoting male same-sex sexual orientation might shift a gay man’s brain to differ more substantially from that of a heterosexual man in the same domain. Simultaneously, the exploration of the participants’ sexual orientation in the current study revealed that women appeared more fluid in their sexual orientation than men. While no heterosexual man could have imagined being in a love relationship with another man ever, but all gay men did, a considerable 21% of the lesbians would not rule out the possibility of being in a love relationship with a man. Even 4% of the heterosexual women were indecisive in this regard. Lesbians further rated their current sexual fantasies as “predominantly homosexual, only incidentally heterosexual,” and not as “exclusively homosexual.” This heightened sexual fluidity might have reduced the possibility for sexual orientation related effects in women.

In women, sexual orientation affects the processing not of emotion-, but sex-related chemosensory signals, with lesbian women probably responding to the relative “novelty” or “unfamiliarity” of male chemosignals. Interestingly, neither did gay men show a similar response to female control sweat (see Table S7), nor did the effect in women transfer to responses to faces. However, the “novelty” of male sweat probably results from fewer physically close encounters to men experienced by lesbian women (Diamond & Dubé, 2002). That gay men did not show a similar pattern is probably related to the fact that close encounters with the other sex are much more common in gay men than in lesbian women (Diamond & Dubé, 2002). In fact, gay men are reported to be unique in their tendency to not affiliate primarily with their own sex (Gillespie et al., 2015), most likely resulting in frequent exposure to female chemosignals. That lesbian women did not show similar specific responses to neutral male faces as they did to male control sweat is probably due to much more frequent encounters with male faces. In contrast to male chemosignals, those are present in the everyday life also of lesbian women, without needing any close contact.

Comparing the results from Studies 1a and 1b with those of Studies 2a and 2b reveals striking differences between the responses to chemosensory vs. visual aggression signals. The effects of sexual orientation on the processing of chemosensory aggression signals are much more prominent compared to the processing of visual aggression signals. In fact, direct effects of sexual orientation are absent in the visual domain, and confidence intervals somewhat question the reliability of the observed effect of only gay men responding differentially to male and female angry expressions (see Table S19). Moreover, apparently the chemosensory aggression signals, when compared to control sweat, are processed as much more significant than angry compared to neutral faces. Apart from methodological considerations as already discussed, this difference might relate to a higher significance of chemosensory signals per se: Several studies combining social signals from the visual and the chemosensory modality have shown that usually the information obtained from chemosensory signals “override” those from the visual modality (Hoenen et al., 2018; Pause et al., 2004; Zernecke et al., 2011). This high impact probably originates from chemosensory signals being inherently honest social signals, as their release is not prone to fabrication or falsification, which is the case with affective facial expressions. In addition to this general significance, sexual orientation might specifically be linked to chemosensory communication via genetic factors underlying both sexual orientation and olfaction: A recent, large-scale genome-wide association study revealed single nucleotid polymorphisms associated with male sexual orientation within a locus containing a high density of olfactory receptor genes (Ganna et al., 2019).

It has to be acknowledged that the data presented here were obtained from relatively small samples. This is due to the fact that the current studies are, to our knowledge, the first of their kind, and thus focused on high internal validity with homogenous samples. This approach yielded a high rate of individuals (roughly 40% in Studies 1a and 1b, and about 60% in Studies 2a and 2b) who could not be included due to not meeting one or more of the extensive inclusion criteria. Even though, the achieved statistical power was satisfactory, and both the effect sizes and the confidence intervals of the mean differences are indicative of substantial effects, most probably not related to sampling errors. Moreover, although the effects observed in the current studies can be considered valid, the studies were not designed to provide a definitive answer to the question of the underlying mechanism. We here proposed that the way in which gay men respond to chemosensory aggression signals is the result of evolutionary processes selecting for heightened social integration (sensu Barron & Hare, 2020). Thus, we assume that these responses are somewhat “biologically hardwired” in gay men, to the same degree as their sexual orientation. There are, however, other possible explanations, one of which relates to a recent proposition by Diamond and Alley (2022). Diamond and Alley suggest that sexual minority populations are subject to a chronic absence of social safety beginning in early childhood, and adapt to this unsafety at various functional levels, for example by developing a hypervigilance to social threat. These adaptations then may result in adverse health effects. Thus, gay men’s responses to male aggression signals could be indicative of such hypervigilance, but the absence of a similar effect in lesbian women would not have been predicted. Our participants did not provide any information regarding childhood adversity or their level of exposure to homonegativity, thus we cannot offer any indicators in favor or against this notion. Of note, however, both the male and the female participants included in the current studies had to be of excellent health, physically as well as mentally. Hence, in case of any adaptions to assumed social insecurity on behalf of the gay men (and the lesbian women), these had at least not yielded adverse health effects. Nonetheless, the model proposed by Diamond and Alley (2022) provides an alternative framework for the discussion of the current results, and while we still consider chemosensory aggression signals as honest signals and their message to be fixed, responses to this message might be shaped by social learning.

Taken together, the current studies demonstrate that sexual orientation, especially in men, affects the processing of social aggression signals. The results are in line with evolutionary theories considering the adaptive value of same-sex sexual attraction within humans. Future studies will have to show whether these effects are restricted to the communication of aggression, or expand to other, possibly more prosocial emotions as well. Further, it remains to be examined if and how the observed differences translate into overt social behavior.

### Supplementary Information

Below is the link to the electronic supplementary material.

Supplementary file1 (DOCX 5516 kb)

## Data Availability

The datasets generated for this study are available upon request from the corresponding author.
