# Supplementary material for: Sexual Orientation Affects Neural Responses to Subtle Social Aggression Signals
Source: Arch Sex Behav. 2023 Jul 27;53(1):153–75. doi: 10.1007/s10508-023-02661-z (PMC10794475; doi:10.1007/s10508-023-02661-z)
Supplement: Supplementary file 1 — Supplementary file1 (DOCX 5516 kb) [file 10508_2023_2661_MOESM1_ESM.docx]

**Sexual Orientation Affects Neural Responses to Subtle Social Aggression Signals**

**Supplementary Material**

PART A: Study 1a & Study 1b, METHODS

PART B: Study 1a, CSERP TOPOGRAPHY

PART C: Study 1a, RESULTS

PART D: Study 1b, CSERP TOPOGRAPHY

PART E: Study 1b, RESULTS

PART F: Study 2a & Study 2b, METHODS

PART G: Study 2a, RESULTS

PART H: Study 2b, RESULTS

References

**PART A: Study 1a & Study 1b, METHODS**

**Stimulus Presentation**

For EEG recording and stimulus ratings, the chemosensory stimuli were presented according to the method described by [Kobal and Hummel (1988)](#_ENREF_2), using a constant-flow (100 ml/s; stimulus duration = 0.4 s) 8-channel olfactometer (OL023, Burghart, Wedel, Germany). Both nostrils were stimulated simultaneously, and both air streams were controlled by separate mass flow meters. In the olfactometer, the glass tubes containing the stimuli were stored in a warm-water chamber, and the odors were delivered to the participants through a Teflon tube. The temperature of the air flow at the exit of the olfactometer was 37 °C and the relative humidity was set above 80%. White noise of 75 dB (A) was presented binaurally over earplugs (ER3-14A Etymotic Research Inc., Elk Grove Village, USA), in order to prevent the participants from hearing the switching valves of the olfactometer. During EEG recordings and odor ratings, participants performed the velopharyngeal closure technique (for review see Lorig, 2000; Pause et al., 1999).

**EEG Procedure**

The procedure of the entire session is presented in figure S1. During EEG recording, 100 stimuli were presented, with 25 presentations of each sweat sample (male aggression, male control, female aggression, female control). The stimuli were presented in a previously randomized, fixed order (with the restriction that the same stimulus was presented no more than twice in a row and the same emotion as well as the donor’s sex no more than three times in a row). Participants were informed that they would receive body odors; however, they did neither know anything about the emotional state of the odor donors, nor how many different odors they would receive. At the beginning of each trial, a fixation cross was presented on a screen for 5.5 s, and the sweat samples were presented for a duration of 0.4 s beginning 2–3 s after cross-onset (randomized). Subsequent to the fixation cross, the screen turned grey for 2–3 s (randomized), followed by the question “Did you smell anything?” appearing on the screen for 3 s. Afterwards, the question “Which was the donor’s sex?” appeared on the screen for 3 s. In order to ensure sustained attention throughout EEG recording in spite of the relatively long ISIs, the participants were further presented with a task during which they had to assign a color to the odor they just had perceived (3 s). The trials ended with the presentation of another grey screen for 2-5 s (randomized). In total, the trials’ duration was 18.5 to 22.5 s (randomized), with a total recording duration of 34:10 min. EEG recordings were subdivided into 3 blocks (33, 33 and 34 trials) separated by 2 individually adjusted resting periods. On average, the EEG procedure lasted 42 min (*SD* = 5 min).

**Figure S1**

*Time Course of the Experimental Session*


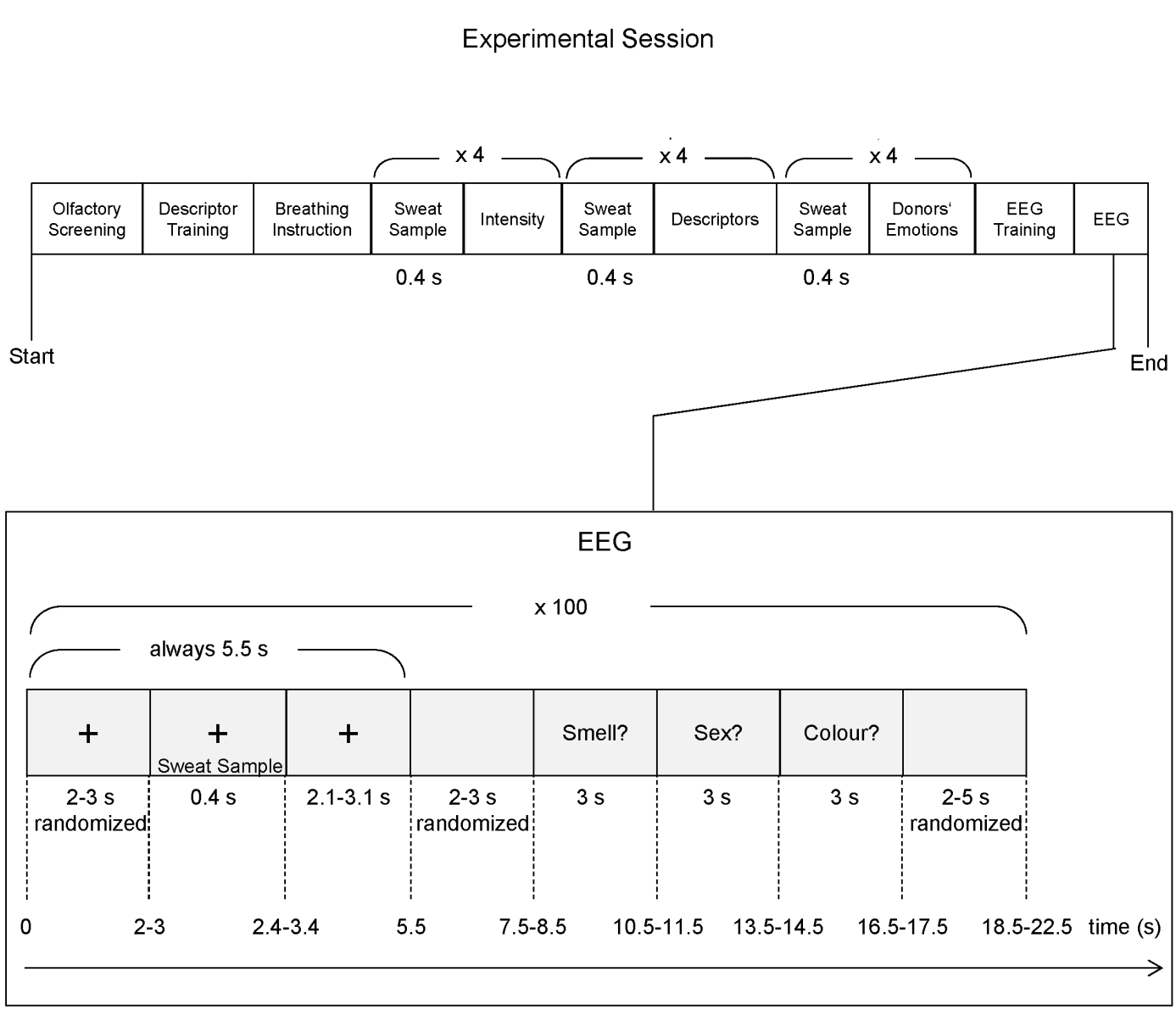


*Note.* First, a brief olfactory screening was carried out. Then the expansive verbal descriptor list was trained and afterwards participants were instructed and trained on the velopharyngeal closure technique. Then, the odor ratings (intensity, verbal descriptors, and donors’ emotions) were obtained. Finally, the EEG and the detection rates were recorded.

**Data Recording and Reduction**

Ongoing EEG was recorded from 61 scalp locations with Ag/AgCl sintered electrodes (inner diameter 6 mm), using an electrode cap (EasyCap GmbH, Herrsching, Germany). For later correction of ocular artefacts, an additional electrode was placed 1.5 cm below the right eye, outside the vertical pupil axis, to record vertical eye movements. Fp2 was used to record the horizontal eye movements. The ground electrode was placed at position FT10. The electrodes’ impedance was usually below 10 and always below 20 kOhm. Data were sampled at 500 Hz with an averaged reference and low-pass filtered online at 135 Hz using a QuickAmp 72 EEG amplifier (Brain Products GmbH, Gilching, Germany). BrainVision Recorder software (Brain Products GmbH, Gilching, Germany) was used to record EEG data.

Offline, EEG signals were re-referenced to linked ear lobes, low pass filtered with 40 Hz (48 dB/octave) and high pass filtered with 0.05 Hz (48 dB/octave). A 50 Hz notch filter was applied. EEG was corrected for eye movements based on the recordings of the horizontal (Fp2) and vertical eye electrodes (Gratton et al., 1983) and baseline-corrected (-500 ms – 0 ms before stimulus onset). Channels containing voltage bursts (i.e. highest allowed difference between minimum and maximum amplitude within an interval of 100 ms: 75 µV, highest and lowest allowed total amplitude from baseline: +/-100 µV) were excluded from analysis. Trials with more than 1/3 channels of one or more pools (see below) contaminated with artefacts were additionally excluded from analysis. Data of 4 participants were completely excluded from analyses due to less than 13 of 25 trials remaining in at least one condition.

For peak detection, signals were low pass filtered with 7 Hz, 48 dB/octave. The 61 scalp electrode positions were subdivided into nine areas (pools) and a mean peak for each pool was calculated by averaging adjacent electrodes in anterior (a), central (c) and posterior (p) areas for the left (l) and the right (r) hemisphere as well as for midline electrodes (m; sagittal line: a, c, p; transversal line: l, m, r; sagittal by transversal: al: AF7, AF3, F7, F5, F3; am: Fpz, AFz,F1, Fz, F2; ar: AF4, AF8, F4, F6, F8; cl: FT7, FC5, FC3, T7, C5, C3, TP7, CP5, CP3; cm: FC1, FCz, FC2, C1, Cz, C2, CP1, CPz, CP2; cr: FC4, FC6, FT8, C4, C6, T8, CP4, CP6, TP8; pl: P7, P5, P3, PO7, PO3, O1; pm: P1, Pz, P2, POz, Oz; pr: P4, P6, P8, PO4, PO8, O2). Because Fp2 was used for recording horizontal eye movements only, Fp1 also was excluded from pooling.

In relation to the baseline period, three separate peaks were detected within predefined latency windows (P2: 500–700 ms, P3-1: 700–900 ms, P3-2: 900-1100 ms, Pause & Krauel, 2000), and amplitudes and latencies of each peak were calculated (also see Pause et al., 2020).

**PART B: Study 1a, CSERP TOPOGRAPHY**

**Topographical Dominance of the CSERP Components**

In order to examine the topographical dominance of the P2, P3-1, and P3-2, respectively, individual peak amplitudes were averaged across stimulus conditions (male aggression, male control, female aggression, female control), and transversal lines (left, midline, right) per each level of the sagittal line. The resulting grand averages were then subjected to an ANOVA with the within-subjects factor Sagittal (SAG: anterior, central, posterior), and follow-up with paired *t*-tests. The results show a posterior dominance of each ERP component as has been reported before (Lübke et al., 2012; Polich, 2007, see tables S1 and S2), and accordingly, the peak amplitudes of the electrodes above posterior scalp regions were subjected to further analyses.

**Table S1**

*Men’s P2, P3-1, and P3-2 Amplitudes across Sagittal Scalp Regions*

|  | P2 | | P3-1 | | P3-2 | |
| --- | --- | --- | --- | --- | --- | --- |
|  | *M* [µV] | *SD* [µV] | *M* [µV] | *SD* [µV] | *M* [µV] | *SD* [µV] |
| anterior | 1.33 | 1.33 | 1.58 | 1.46 | 1.40 | 1.57 |
| central | 2.31 | 2.34 | 3.24 | 2.90 | 3.14 | 2.87 |
| posterior | 2.63 | 2.91 | 3.86 | 3.77 | 3.77 | 3.48 |

**Table S2**

*Tests of the Topographical Dominance of the P2, P3-1 and P3-2 Amplitude in Men*

|  | P2 | P3-1 | P3-2 |
| --- | --- | --- | --- |
| ANOVA | *F*(2, 78) = 14.22, *p* < .001, *η^2^_p_* = .27, Power = 1.00 | *F*(2, 78) = 23.80, *p* < .001, *η^2^_p_* = .38, Power = 1.00 | *F*(2, 78) = 28.33, *p* < .001, *η^2^_p_* = .42, Power = 1.00 |
|  | posterior > central(*) | posterior > central** | posterior > central** |
| Follow-up *t*-tests | posterior > anterior*** | posterior > anterior*** | posterior > anterior*** |
|  | central > anterior*** | central > anterior*** | central > anterior*** |

**PART C: Study 1a, RESULTS**

**Stimulus Detection and Assessment of Donors’ Sex**

**Table S3**

*Men’s (n = 40) Stimulus Detection*

| Group | Male aggression sweat | | Male control sweat | | Female aggression sweat | | Female control sweat | |
| --- | --- | --- | --- | --- | --- | --- | --- | --- |
|  | *M* [%] | *SD* [%] | *M* [%] | *SD* [%] | *M* [%] | *SD* [%] | *M* [%] | *SD* [%] |
| Gay Men | 58.59 | 30.79 | 52.47 | 25.73 | 49.18 | 29.59 | 47.77 | 28.76 |
| Heterosexual Men | 57.74 | 30.34 | 55.13 | 26.50 | 49.39 | 27.91 | 51.13 | 29.22 |
| All Men | 58.10 | 30.14 | 54.00 | 25.88 | 49.30 | 28.26 | 49.70 | 28.50 |

**Table S4**

*Men’s (n = 40) Assessments of the Donors’ Sex*

| Group | Male aggression sweat | | Male control sweat | | Female aggression sweat | | Female control sweat | |
| --- | --- | --- | --- | --- | --- | --- | --- | --- |
|  | *M* [%] | *SD* [%] | *M* [%] | *SD* [%] | *M* [%] | *SD* [%] | *M* [%] | *SD* [%] |
| Gay Men | 58.45 | 18.58 | 55.79 | 19.28 | 48.46 | 14.47 | 45.89 | 20.72 |
| Heterosexual Men | 50.44 | 19.46 | 44.57 | 16.65 | 52.82 | 17.51 | 55.20 | 16.52 |
| All Men | 53.84 | 19.27 | 49.34 | 18.45 | 50.97 | 16.24 | 51.24 | 18.76 |

**Odor Ratings and Descriptions**

**Table S5**

*Men’s (n = 39) Odor Intensity Ratings*

| Group | Male aggression sweat | | Male control sweat | | Female aggression sweat | | Female control sweat | |
| --- | --- | --- | --- | --- | --- | --- | --- | --- |
|  | *M* | *SD* | *M* | *SD* | *M* | *SD* | *M* | *SD* |
| Gay Men | 3.56 | 2.07 | 3.81 | 1.60 | 2.81 | 1.47 | 2.88 | 1.75 |
| Heterosexual Men | 3.70 | 2.23 | 3.17 | 2.04 | 3.04 | 1.92 | 2.61 | 1.62 |
| All Men | 3.64 | 2.13 | 3.44 | 1.88 | 2.95 | 1.73 | 2.72 | 1.65 |

*Note*: Range: 1-9.

**Table S6**

*Men’s Suspicion of the Donors’ Affective State*

| Assessed  Emotion | Male aggression sweat  (*n* = 37) | | Male control sweat  (*n* = 38) | | Female aggression sweat  (*n* = 38) | | Female control sweat  (*n* = 38) | |
| --- | --- | --- | --- | --- | --- | --- | --- | --- |
|  | *M* | *SD* | *M* | *SD* | *M* | *SD* | *M* | *SD* |
| Anger | 2.30 | 2.15 | 1.44 | 1.63 | 1.66 | 1.59 | 1.75 | 2.13 |
| Fear | 2.15 | 2.30 | 1.49 | 1.42 | 2.19 | 2.22 | 1.62 | 2.12 |
| Happiness | 1.41 | 1.79 | 2.26 | 2.56 | 2.15 | 2.38 | 2.03 | 2.39 |

*Note*: Range: 0-10.

**Figure S2**

*Frequency of Verbal Descriptors Assigned to Male Sweat Samples by Men*


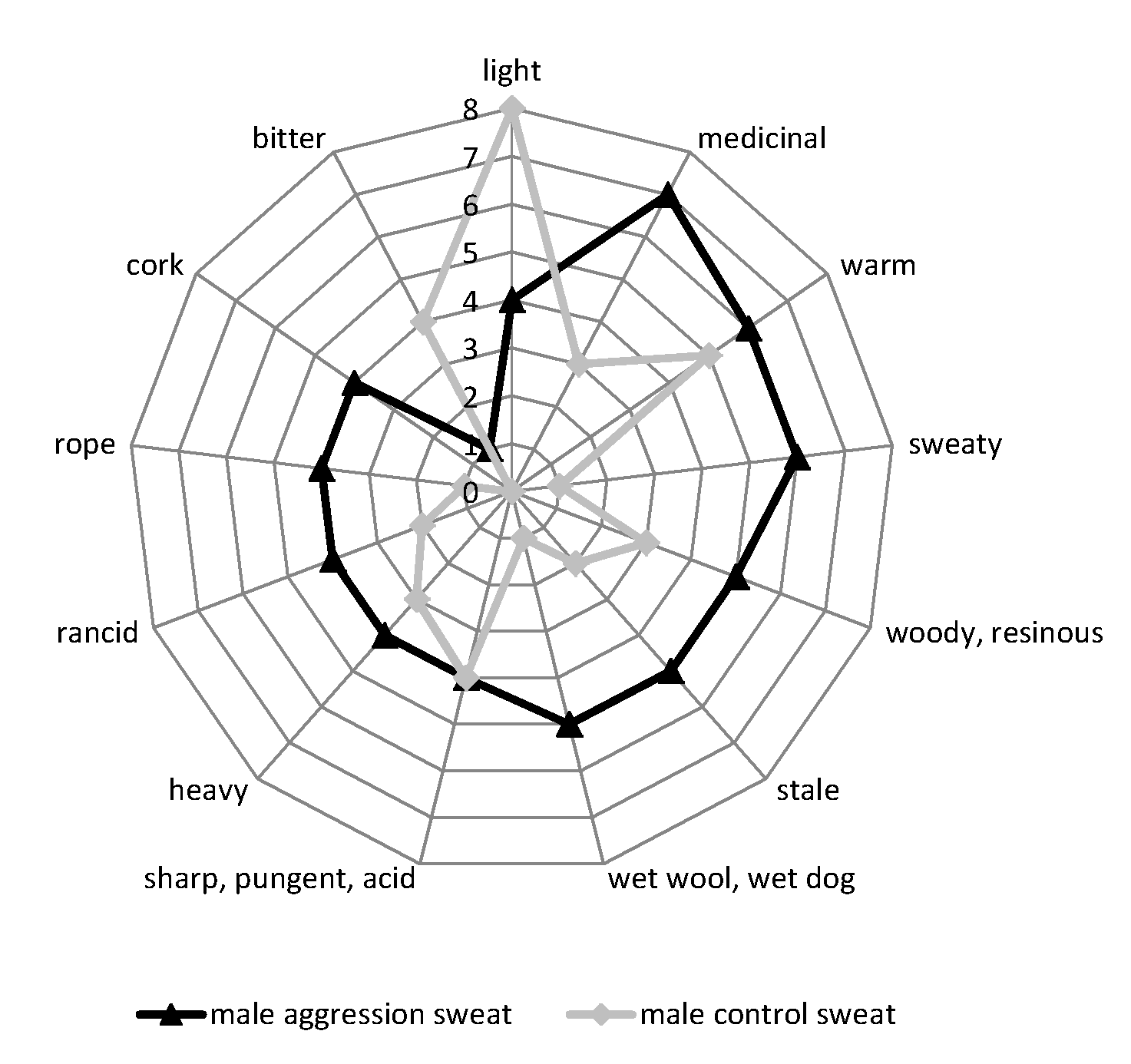


*Note.* Only descriptors chosen at least 4 times to describe one of the samples are depicted.

**Figure S3**

*Frequency of Verbal Descriptors Assigned to Female Sweat Samples by Men*


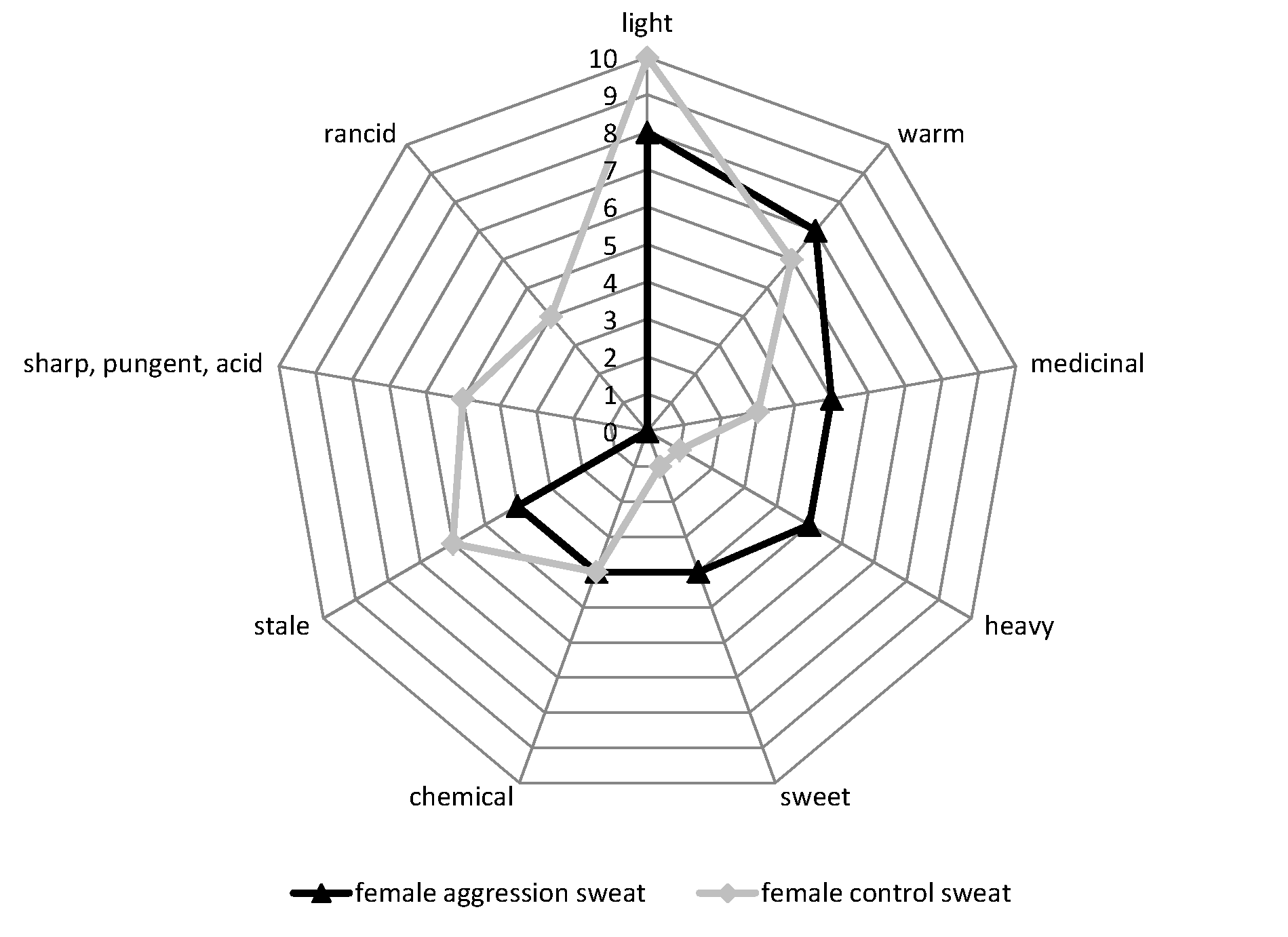


*Note.* Only descriptors chosen at least 4 times to describe one of the samples are depicted.

**Confidence intervals of effects within the CSERP**

**Table S7**

*Mean Differences and 95% Confidence Intervals of Effects within the P2, P3-1 and P3-2 Amplitudes in Men*

| Peak | ANOVA | (Simple) Effect | *MD* | *SED* | 95% *CI* |
| --- | --- | --- | --- | --- | --- |
| P2 | SO | GM > HM* | 2.23 | 0.87 | [0.47, 3.99] |
|  | DS | MS > FS* | 0.71 | 0.35 | [0.14, 1.41] |
|  | SO x EMO x DS | GM > HM in MS in CS^n.s.^ | 1.79 | 1.13 | [-0.50, 4.07] |
|  |  | AS > CS in MS in HM^n.s.^ | 0.04 | 0.73 | [-1.48, 1.55] |
|  |  | MS > FS in CS in GM^n.s.^ | 0.26 | 0.47 | [-0.73, 1.25] |
| P3-1 | SO | GM > HM* | 2.98 | 1.12 | [0.72, 5.25] |
|  | DS | MS > FS* | 0.81 | 0.37 | [0.06, 1.55] |
|  | EMO | AS > CS^n.s.^ | 0.43 | 0.32 | [-0.23, 1.08] |
|  | SO x EMO x DS | GM > HM in MS in AS** | 3.90 | 1.31 | [1.25, 6.55] |
|  |  | AS > CS in MS in GM** | 1.84 | 0.47 | [0.86, 2.83] |
|  |  | MS > FS in AS in GM** | 1.87 | 0.73 | [0.33, 3.41] |
| P3-2 | SO | GM > HM* | 2.69 | 1.04 | [0.58, 4.79] |
|  | DS | MS > FS* | 0.97 | 0.39 | [0.18, 1.76] |
|  | EMO | AS > CS* | 0.80 | 0.30 | [0.19, 1.41] |

*Note*: *MD* = Mean Difference, *SED* = Standard Error of the Difference, *CI* = Confidence Interval, SO = Sexual Orientation: GM = gay men, HM = heterosexual men, EMO = Emotion: AS = anger sweat, CS = control sweat, DS = Donors’ Sex: MS = male sweat, FS = female sweat.

**p* ≤ .05. ***p* < .01. ^n.s.^ = ANOVA or nested effects analyses not significant (*p* > .05).

**Table S8**

*Mean Differences and 95% Confidence Intervals of Effects within the P2, P3-1 and P3-2 Latencies in Men*

| Peak | ANOVA | (Simple) Effect | *MD* | *SED* | 95% *CI* |
| --- | --- | --- | --- | --- | --- |
| P2 | DS | MS > FS** | 23.47 | 8.18 | [6.92, 40.01] |
| P3-1 | SO x EMO | GM > HM in CS^n.s.^ | 3.69 | 12.35 | [-21.32, 28.70] |
|  |  | AS > CS in HM^n.s.^ | 5.35 | 9.16 | [-13.66, 24.35] |
| P3-2 | EMO x DS | FS > MS in CS* | 26.28 | 10.62 | [4.80, 47.77] |

*Note*: *MD* = Mean Difference, *SED* = Standard Error of the Difference, *CI* = Confidence Interval, SO = Sexual Orientation: GM = gay men, HM = heterosexual men, EMO = Emotion: AS = anger sweat, CS = control sweat DS = Donors’ Sex: MS = male sweat, FS = female sweat.

***p* < .01. ^n.s.^ = ANOVA or nested effects analyses not significant (*p* > .05).

**PART D: Study 1b, CSERP TOPOGRAPHY**

**Topographical Dominance of the CSERP Components**

In order to examine the topographical dominance of the P2, P3-1, and P3-2, respectively, individual peak amplitudes were averaged across stimulus conditions (male aggression, male control, female aggression, female control), and transversal lines (left, midline, right) per each level of the sagittal line. The resulting grand averages were then subjected to an ANOVA with the within-subjects factor Sagittal (SAG: anterior, central, posterior) and follow-up paired *t*-tests. The results show a posterior dominance of each ERP component as has been reported before (Lübke et al., 2012; Polich, 2007, see tables S9 and S10), and accordingly, the peak amplitudes of the electrodes above posterior scalp regions were subjected to further analyses.

**Table S9**

*Women’s P2, P3-1, and P3-2 Amplitudes across Sagittal Scalp Regions*

|  | P2 | | P3-1 | | P3-2 | |
| --- | --- | --- | --- | --- | --- | --- |
|  | *M* [µV] | *SD* [µV] | *M* [µV] | *SD* [µV] | *M* [µV] | *SD* [µV] |
| anterior | 1.28 | 1.02 | 1.74 | 1.23 | 1.59 | 1.14 |
| central | 2.15 | 1.46 | 3.22 | 1.97 | 3.04 | 1.97 |
| posterior | 2.47 | 2.91 | 3.81 | 2.58 | 3.55 | 2.62 |

**Table S10**

*Tests of the Topographical Dominance of the P2, P3-1 and P3-2 Amplitude in Women*

|  | P2 | P3-1 | P3-2 |
| --- | --- | --- | --- |
| ANOVA | *F*(2, 86) = 26.14, *p* < .001, *η^2^_p_* = .38, Power = 1.00 | *F*(2, 86) = 42.51, *p* < .001, *η^2^_p_* = .50, Power = 1.00 | *F*(2, 86) = 35.78, *p* < .001, *η^2^_p_* = .45, Power = 1.00 |
|  | posterior > central* | posterior > central** | posterior > central* |
| Follow-up *t*-tests | posterior > anterior*** | posterior > anterior*** | posterior > anterior*** |
|  | central > anterior*** | central > anterior*** | central > anterior*** |

*Note*: * *p* < .05. ** *p* < .01. *** *p* < .001.

**PART E: Study 1b, RESULTS**

**Stimulus Detection and Assessment of Donors’ Sex**

**Table S11**

*Women’s (n = 44) Stimulus Detection*

| Group | Male aggression sweat | | Male control sweat | | Female aggression sweat | | Female control sweat | |
| --- | --- | --- | --- | --- | --- | --- | --- | --- |
|  | *M* [%] | *SD* [%] | *M* [%] | *SD* [%] | *M* [%] | *SD* [%] | *M* [%] | *SD* [%] |
| Lesbian Women | 64.42 | 23.47 | 46.74 | 24.19 | 48.63 | 27.04 | 46.11 | 26.98 |
| Heterosexual Women | 62.08 | 30.14 | 49.28 | 29.32 | 50.40 | 30.22 | 43.52 | 27.04 |
| All Women | 63.09 | 27.18 | 48.18 | 26.95 | 49.64 | 28.58 | 44.64 | 26.73 |

**Table S12**

*Women’s (n = 44) Assessments of the Donors’ Sex*

| Group | Male aggression sweat | | Male control sweat | | Female aggression sweat | | Female control sweat | |
| --- | --- | --- | --- | --- | --- | --- | --- | --- |
|  | *M* [%] | *SD* [%] | *M* [%] | *SD* [%] | *M* [%] | *SD* [%] | *M* [%] | *SD* [%] |
| Lesbian Women | 62.33 | 16.43 | 52.17 | 17.70 | 44.97 | 15.66 | 51.57 | 18.92 |
| Heterosexual Women | 54.42 | 14.38 | 50.60 | 19.92 | 53.50 | 13.10 | 51.03 | 14.80 |
| All Women | 57.83 | 15.63 | 51.28 | 18.80 | 49.82 | 14.72 | 51.27 | 16.50 |

**Odor Ratings and Descriptions**

**Table S13**

*Women’s (n = 43) Odor Intensity Ratings*

| Group | Male aggression sweat | | Male control sweat | | Female aggression sweat | | Female control sweat | |
| --- | --- | --- | --- | --- | --- | --- | --- | --- |
|  | *M* | *SD* | *M* | *SD* | *M* | *SD* | *M* | *SD* |
| Lesbian Women | 3.94 | 2.65 | 2.50 | 1.34 | 3.50 | 2.31 | 2.83 | 1.92 |
| Heterosexual Women | 3.60 | 2.29 | 2.88 | 1.90 | 2.40 | 1.94 | 2.76 | 1.92 |
| All Women | 3.74 | 2.42 | 2.72 | 1.68 | 2.86 | 2.15 | 2.79 | 1.90 |

*Note*: Range: 1-9.

**Table S14**

*Women’s Suspicion of the Donors’ Affective State*

| Assessed  Emotion | Male aggression sweat  (*n* = 43) | | Male control sweat  (*n* = 39) | | Female aggression sweat  (*n* = 43) | | Female control sweat  (*n* = 42) | |
| --- | --- | --- | --- | --- | --- | --- | --- | --- |
|  | *M* | *SD* | *M* | *SD* | *M* | *SD* | *M* | *SD* |
| Anger | 2.15 | 2.24 | 2.27 | 2.17 | 1.58 | 1.86 | 1.79 | 1.92 |
| Fear | 3.36 | 3.12 | 2.62 | 2.34 | 1.91 | 1.95 | 1.60 | 1.75 |
| Happiness | 1.46 | 1.71 | 2.21 | 2.61 | 2.59 | 2.63 | 2.51 | 2.50 |

*Note*: Range: 0-10.

**Figure S4**

*Frequency of Verbal Descriptors Assigned to Male Sweat Samples by Women*


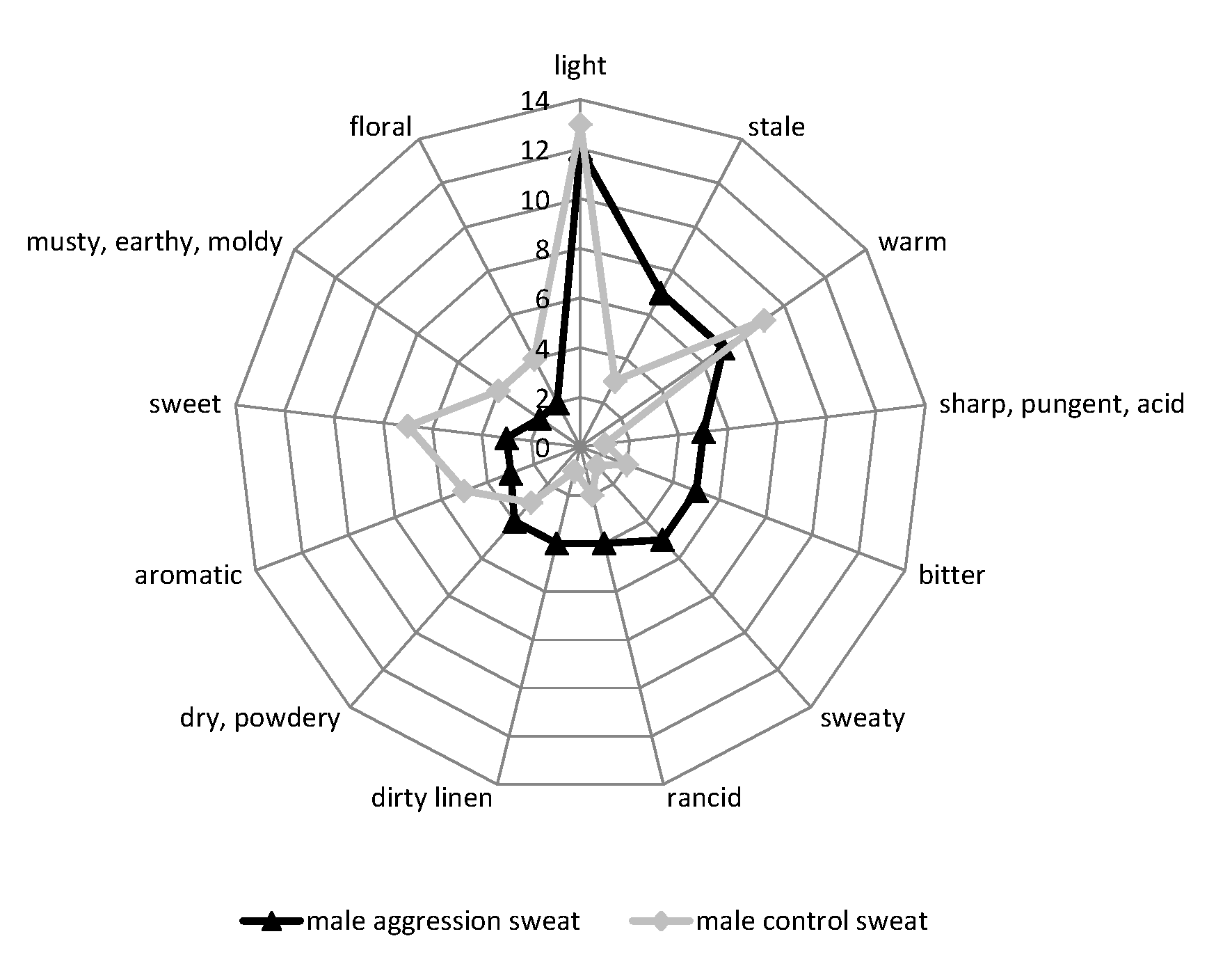


*Note.* Only descriptors chosen at least 4 times to describe one of the samples are depicted.

**Figure S5**

*Frequency of Verbal Descriptors Assigned to Female Sweat Samples by Women*


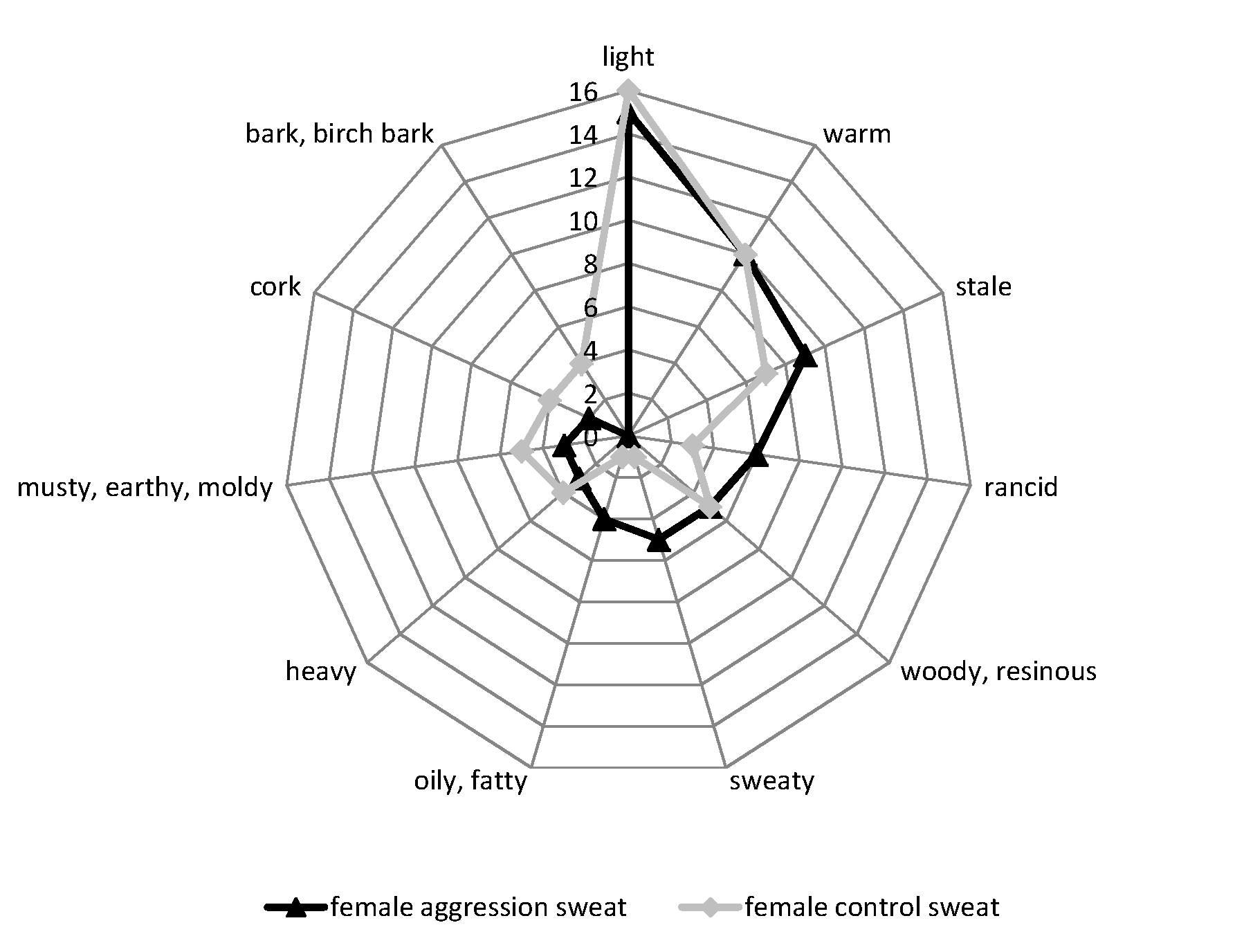


*Note.* Only descriptors chosen at least 4 times to describe one of the samples are depicted.

**Confidence intervals of effects within the CSERP**

**Table S15**

*Mean Differences and 95% Confidence Intervals of Effects within the P2, P3-1 and P3-2 Amplitudes in Women*

| Peak | ANOVA | (Simple) Effect | *MD* | *SED* | 95% *CI* |
| --- | --- | --- | --- | --- | --- |
| P2 | SO | LW > HW^n.s.^ | 0.34 | 0.59 | [-0.85, 1.52] |
|  | DS | MS > FS^n.s.^ | 0.44 | 0.37 | [-0.30, 1.18] |
|  | SO x EMO x DS | LW > HW in MS in CS* | 1.54 | 0.61 | [0.31, 2.76] |
|  |  | AS > CS in MS in HW* | 1.26 | 0.58 | [0.07, 2.46] |
|  |  | MS > FS in CS in LW* | 1.47 | 0.60 | [0.20, 2.73] |
| P3-1 | SO | LW > HW^n.s.^ | 0.16 | 0.79 | [-1.45, 1.76] |
|  | DS | MS > FS** | 1.19 | 2.54 | [0.42, 1.97] |
|  | EMO | AS > CS* | 0.80 | 0.37 | [0.05, 1.54] |
|  | SO x EMO x DS | LW > HW in MS in AS^n.s.^ | 0.33 | 1.33 | [-2.37, 3.02] |
|  |  | AS > CS in MS in LW^n.s.^ | 0.81 | 0.79 | [-0.85, 2.46] |
|  |  | MS > FS in AS in LW^n.s.^ | 1.75 | 0.71 | [0.26, 3.24] |
| P3-2 | SO | LW > HW^n.s.^ | 0.05 | 0.81 | [-1.58, 1.68] |
|  | DS | MS > FS*** | 1.50 | 0.42 | [0.66, 2.35] |
|  | EMO | AS > CS^n.s.^ | 0.70 | 0.42 | [-0.15, 1.55] |

*Note*: *MD* = Mean Difference, *SED* = Standard Error of the Difference, *CI* = Confidence Interval, SO = Sexual Orientation: LW = lesbian women, HM = heterosexual women, EMO = Emotion: AS = anger sweat, CS = control sweat, DS = Donors’ Sex: MS = male sweat, FS = female sweat.

**p* ≤ .05. ***p* < .01. ****p* < .001. ^n.s.^ = ANOVA or nested effects analyses not significant (*p* > .05).

**Table S16**

*Mean Differences and 95% Confidence Intervals of Effects within the P2, P3-1 and P3-2 Latencies in Women*

| Peak | ANOVA | (Simple) Effect | *MD* | *SED* | 95% *CI* |
| --- | --- | --- | --- | --- | --- |
| P2 | DS | MS > FS^n.s.^ | 0.83 | 10.44 | [-20.23, 21.88] |
| P3-1 | SO x EMO | LW > HW in CS* | 27.08 | 12.22 | [2.43, 51.74] |
|  |  | AS > CS in HW* | 20.45 | 9.49 | [0.89, 40.02] |
| P3-2 | EMO x DS | FS > MS in CS^n.s.^ | 6.88 | 11.84 | [-17.01, 30.77] |

*Note*: *MD* = Mean Difference, *SED* = Standard Error of the Difference, *CI* = Confidence Interval, SO = Sexual Orientation: LW = lesbian women, HM = heterosexual women, EMO = Emotion: AS = anger sweat, CS = control sweat, DS = Donors’ Sex: MS = male sweat, FS = female sweat.

**p* ≤ .05. ^n.s.^ = ANOVA or nested effects analyses not significant (*p* > .05).

**PART F: Study 2a & Study 2b, METHODS**

**Figure S6**

*Examples of Pictures of Facial Expressions Presented in Studies 2a and 2b*


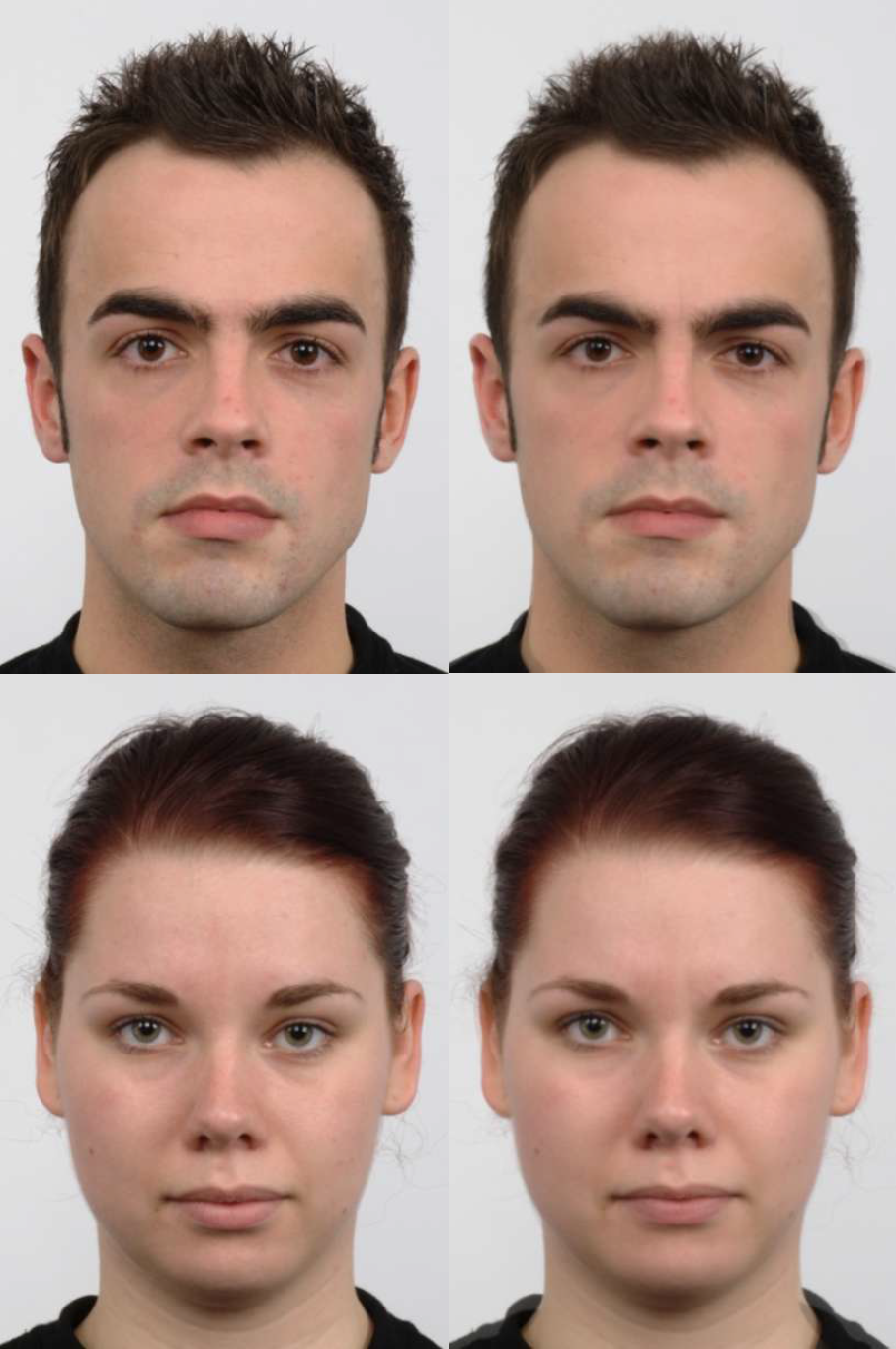


*Note:* Neutral (left column) and angry (right column, 20%) facial expressions displayed by a male (upper row) and a female (lower row) model of the Radboud Faces Database (Langner et al., 2010).

**EEG Procedure**

During EEG recording, 80 stimuli were presented, with 20 presentations of each class of stimuli (male anger face, male neutral face, female anger face, female neutral face). Each of the individual pictures was presented four times. The stimuli were presented in a previously randomized, fixed order (with the restriction that the each of the 10 models was presented no more than twice in succession and the same emotion no more than three times in succession). At the beginning of each trial, a grey screen was presented for 2 s. Afterwards, a fixation cross was displayed for 1.5–2.5 s (randomized), followed by the picture of a neutral or angry face (0.5 s). Subsequent to the picture, a grey screen was shown again for 2–3 s, (randomized), followed by the presentation of the visual analogue scales for the emotional rating for 12 s. Within this timeframe, participants indicated their judgment of the intensity of each of the three basic emotions contained within the facial expression by setting a mark on each visual analogue scale with the mouse. Afterwards, a new trial started. In total, a trial’s duration was 18 to 20 s (randomized). EEG recordings were subdivided into 2 blocks (á 40 trials), separated by a resting period, the duration of which was individually adjusted to each participant. On average, the EEG part, including the break, lasted 28 min (*SD* = 2 min).

**PART G: Study 2a, RESULTS**

**Table S17**

*Men’s Intensity Ratings of Emotions Inferred from Neutral and Angry Faces (Assessed Emotion)*

|  | Angry Male Face | | Neutral Male Face | | Angry Female Face | | Neutral Female Face | |
| --- | --- | --- | --- | --- | --- | --- | --- | --- |
|  | *M* | *SD* | *M* | *SD* | *M* | *SD* | *M* | *SD* |
| anger | 2.34 | 1.38 | 1.79 | 1.40 | 2.30 | 1.45 | 1.69 | 1.23 |
| fear | 1.35 | 1.27 | 1.46 | 1.41 | 1.42 | 1.11 | 1.43 | 1.09 |
| happiness | 0.67 | 0.82 | 0.72 | 0.93 | 0.78 | 0.78 | 0.82 | 0.82 |

**Table S18**

*Differences between Men’s Intensity Ratings of Emotions Inferred from Neutral and Angry Faces (Assessed Emotion)*

|  | Angry Male Face | Neutral Male Face | Angry Female Face | Neutral Female Face |
| --- | --- | --- | --- | --- |
| ANOVA | *F*(2, 82) = 49.23, *p* < .001, *η^2^_p_* = .55, Power = 1.00 | *F*(2, 82) = 18.98, *p* < .001, *η^2^_p_* = .32, Power = 1.00 | *F*(2, 82) = 43.54, *p* < .001, *η^2^_p_* = .52, Power = 1.00 | *F*(2, 82) = 20.29, *p* < .001, *η^2^_p_* = .33, Power = 1.00 |
|  | anger > fear*** | anger > fear(*) | anger > fear*** | anger > fear(*) |
| Follow-up t-tests | anger > happiness*** | anger > happiness*** | anger > happiness*** | anger > happiness*** |
|  | fear > happiness*** | fear > happiness*** | fear > happiness*** | fear > happiness*** |

*Note*: (*) *p* < .10. *** *p* < .001.

**Figure S7**

*Grand Averages of Men’s Visual Event-Related Potentials (VERPs)*


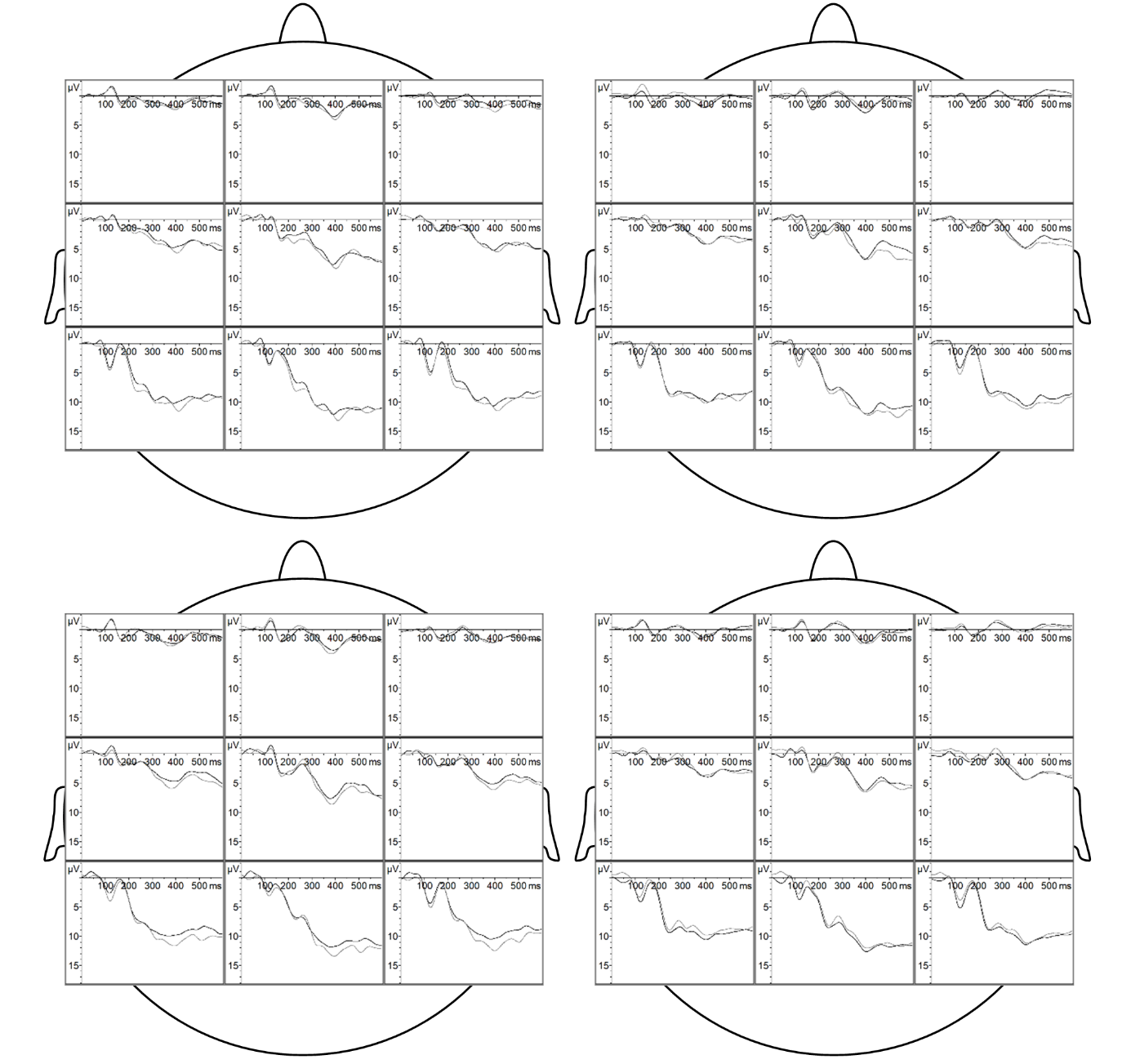


*Note.* Grand averages of the visual event-related potentials (VERPs) across gay men (left column) and heterosexual men (right column) in response to male (upper row) and female (lower row) faces. Black lines indicate VERPs to angry faces, and grey lines indicate VERPs to neutral sweat. Time point 0 refers to the picture onset.

**Table S19**

*Mean Differences and 95% Confidence Interval of the Effect within the N170 Latency in Men*

| Peak | ANOVA | (Simple) Effect | *MD* | *SED* | 95% *CI* |
| --- | --- | --- | --- | --- | --- |
| N170 | SO x EMO x FS | MF > FF in AF in GM* | 9.14 | 4.95 | [-1.19, 19.47] |

*Note*: *MD* = Mean Difference, *SED* = Standard Error of the Difference, *CI* = Confidence Interval, FS = Faces’ Sex: MF = male face, FF = female face, SO = Sexual Orientation: GM = gay men, HM = heterosexual men, EMO = Emotion: AF = angry face, NF = neutral face.

**p* < .05.

**PART H: Study 2b, RESULTS**

**Table S20**

*Women’s Intensity Ratings of Emotions Inferred from Neutral and Angry Faces (Assessed Emotion)*

|  | Angry Male Face | | Neutral Male Face | | Angry Female Face | | Neutral Female Face | |
| --- | --- | --- | --- | --- | --- | --- | --- | --- |
|  | *M* | *SD* | *M* | *SD* | *M* | *SD* | *M* | *SD* |
| anger | 2.75 | 1.64 | 1.93 | 1.61 | 2.74 | 1.51 | 1.99 | 1.61 |
| fear | 1.62 | 1.55 | 1.71 | 1.60 | 1.50 | 1.51 | 1.59 | 1.59 |
| happiness | 0.64 | 0.64 | 0.76 | 0.79 | 0.86 | 0.77 | 1.02 | 0.71 |

**Table S21**

*Differences between Women’s Intensity Ratings of Emotions Inferred from Neutral and Angry Faces (Assessed Emotion)*

|  | Angry Male Face | Neutral Male Face | Angry Female Face | Neutral Female Face |
| --- | --- | --- | --- | --- |
| ANOVA | *F*(2, 86) = 69.49, *p* < .001,  *η^2^_p_* = .62, Power = 1.00 | *F*(2, 86) = 23.26, *p* < .001,  *η^2^_p_* = .35, Power = 1.00 | *F*(2, 86) = 50.30, *p* < .001,  *η^2^_p_* = .54, Power = 1.00 | *F*(2, 86) = 15.38, *p* < .001,  *η^2^_p_* = .26, Power = 1.00 |
|  | anger > fear*** | n.s. | anger > fear*** | anger > fear** |
| Follow-up t-tests | anger > happiness*** | anger > happiness*** | anger > happiness*** | anger > happiness*** |
|  | fear > happiness*** | fear > happiness*** | fear > happiness** | fear > happiness** |

*Note*: (*): *p* < .10. **: *p* < .01. ***: *p* < .001. n.s.: non-significant.

**Figure S8**

*Grand Averages of Women’s Visual Event-Related Potentials (VERPs)*


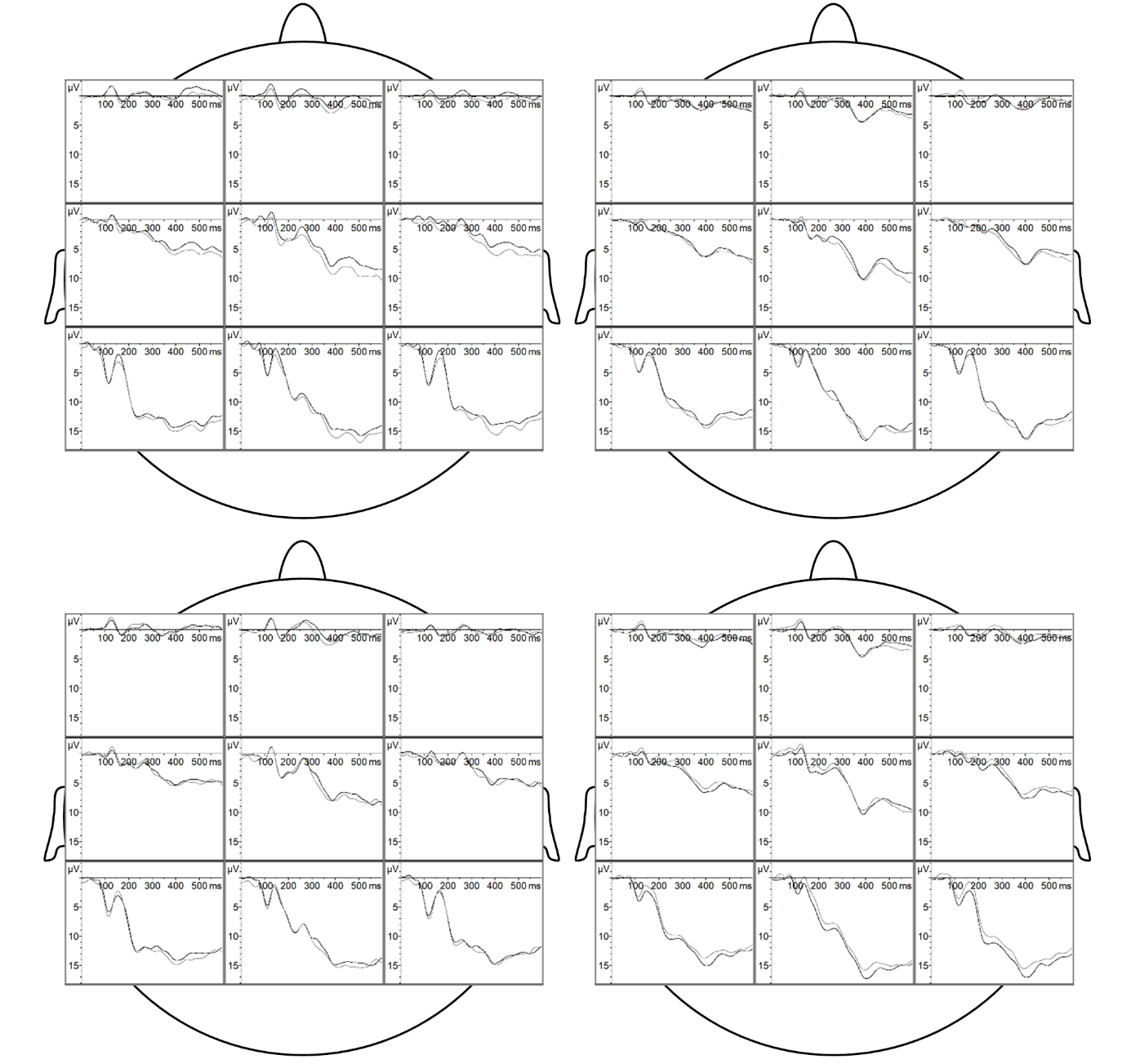


*Note.* Grand Averages of the visual event-related potentials (VERPs) across lesbian women (left column) and heterosexual women (right column) in response to male (upper row) and female (lower row) faces. Black lines indicate VERPs to angry faces, and grey lines indicate VERPs to neutral sweat. Time point 0 refers to the picture onset.

**Table S22**

*Mean Differences and 95% Confidence Interval of the Effect within the N170 Latency in Women*

| Peak | ANOVA | (Simple) Effect | *MD* | *SED* | 95% *CI* |
| --- | --- | --- | --- | --- | --- |
| N170 | SO x EMO x FS | MF > FF in AF in LW^n.s.^ | 3.93 | 4.97 | [-6.51, 14.37] |

*Note*: *MD* = Mean Difference, *SED* = Standard Error of the Difference, *CI* = Confidence Interval, FS = Faces’ Sex: MF = male face, FF = female face, SO = Sexual Orientation: LW = lesbian women, HW = heterosexual women, EMO = Emotion: AF = angry face, NF = neutral face.

n.s. = ANOVA or nested effects analyses not significant (*p* > .05).

**References**

Gratton, G., Coles, M. G., & Donchin, E. (1983). A new method for off-line removal of ocular artifact. *Electroencephalography and Clinical Neurophysiology, 55*(4), 468-484. https://doi.org/10.1016/0013-4694(83)90135-9

Kobal, G., & Hummel, C. (1988). Cerebral chemosensory evoked potentials elicited by chemical stimulation of the human olfactory and respiratory nasal mucosa. *Electroencephalography and Clinical Neurophysiology/Evoked Potentials Section, 71*(4), 241-250. https://doi.org/10.1016/0168-5597(88)90023-8

Langner, O., Dotsch, R., Bijlstra, G., Wigboldus, D. H. J., Hawk, S. T., & van Knippenberg, A. (2010). Presentation and validation of the Radboud Faces Database. *Cognition and Emotion, 24*(8), 1377-1388. https://doi.org/10.1080/02699930903485076

Lorig, T. S. (2000). The application of electroencephalographic techniques to the study of human olfaction: A review and tutorial. *International Journal of Psychophysiology, 36*(2), 91-104. https://doi.org/10.1016/s0167-8760(99)00104-x

Lübke, K. T., Hoenen, M., & Pause, B. M. (2012). Differential processing of social chemosignals obtained from potential partners in regards to gender and sexual orientation. *Behavioural Brain Research, 228*, 375-387. https://doi.org/10.1016/j.bbr.2011.12.018

Pause, B. M., & Krauel, K. (2000). Chemosensory event-related potentials (CSERP) as a key to the psychology of odors. *International Journal of Psychophysiology, 36*(2), 105-122. https://doi.org/10.1016/S0167-8760(99)00105-1

Pause, B. M., Krauel, K., Sojka, B., & Ferstl, R. (1999). Is odor processing related to oral breathing? *International Journal of Psychophysiology, 32*(3), 251-260. https://doi.org/10.1016/S0167-8760(99)00020-3

Pause, B. M., Storch, D., & Lübke, K. T. (2020). Chemosensory communication of aggression: Women's fine-tuned neural processing of male aggression signals. *Philosophical Transactions of the Royal Society B-Biological Sciences, 375*(1800), 20190270. https://doi.org/10.1098/rstb.2019.0270

Polich, J. (2007). Updating P300: An integrative theory of P3a and P3b. *Clinical Neurophysiology, 118*(10), 2128-2148. https://doi.org/10.1016/j.clinph.2007.04.019
